# Type 2 diabetes prediction without labs: a systems-level neural framework for risk and behavioral network reorganization

**DOI:** 10.3389/fdgth.2025.1714545

**Published:** 2026-01-16

**Authors:** Mahreen Kiran, Ying Xie, Graham Ball, Nasreen Anjum, Rudolph Schutte, Barbara Pierscionek

**Affiliations:** 1 Faculty of Health, Medicine and Social Care, Anglia Ruskin University, Chelmsford, United Kingdom; 2 Faculty of Business and Management, Cranfield University, Cranfield, United Kingdom; 3 Intelligent Omics Ltd, Nottingham, United Kingdom; 4 School of Computing, University of Portsmouth, Portsmouth, United Kingdom

**Keywords:** behavioral coherence breakdown, early disease prediction, machine learning, neural network modeling, psychosocial risk factors, survival analysis, Type 2 Diabetes Mellitus (T2DM)

## Abstract

**Background:**

Prediction models for Type 2 Diabetes Mellitus (T2DM) often rely on biochemical markers such as glycated hemoglobin, fasting glucose, or lipid profiles. While clinically informative, these indicators typically reflect established dysglycemia, limiting their value for early prevention. In contrast, psychosocial stress, sleep disturbance, tobacco use, and dietary quality represent modifiable, non-clinical factors that can be observed long before metabolic abnormalities are clinically detectable. Yet most studies examine these factors in isolation or as additive lifestyle scores, overlooking how their interdependencies reorganize in the preclinical phase. A systems-level approach is therefore needed to capture how disruptions in behavioral coherence signal emerging vulnerability.

**Methods:**

This study develops a dual-analytic framework that integrates Cox proportional hazards models with artificial neural network (ANN) coherence analysis. Using longitudinal data from the UK Biobank (*n*=15,774; follow-up up to 17 years), we identified non-clinical predictors of incident T2DM and examined how behavioral networks reorganize across health states. Predictors were screened through multivariate survival analysis and mapped into ANN-derived influence matrices to quantify stability, direction, and systemic coherence of relationships among diet, sleep, psychosocial states, and demographics.

**Results:**

Eighteen significant predictors of T2DM onset were identified. Elevated risk was linked to loneliness, psychiatric consultation, emotional distress, insomnia, irregular sleep, tobacco use, and high intake of processed meat, beef, and refined grains. Protective effects were observed for 7–8 h of sleep, oat and muesli consumption, and fermented dairy. ANN analyses revealed a pronounced breakdown of behavioral coherence in T2DM: foods that stabilized mood in healthy individuals became associated with distress, age and BMI lost their anchoring roles, and emotional states emerged as dominant but erratic drivers of diet. These reversals and destabilizations were consistent across model iterations, suggesting robust signatures of preclinical vulnerability.

**Conclusion:**

T2DM risk is better conceptualized as systemic reorganization within behavioral networks rather than the additive effects of isolated factors. By combining survival models with ANN-derived coherence mapping, this study demonstrates that early prediction is possible from modifiable, everyday behaviors without laboratory measures. The framework highlights leverage points for psychologically informed, personalized prevention strategies.

## 1 Introduction

Type 2 Diabetes Mellitus (T2DM) is a major global health threat. Hundreds of millions of adults are affected today and the number is projected to rise sharply in coming decades, with a substantial toll in deaths and health spending (1). If not diagnosed or well managed, T2DM leads to serious complications that include kidney disease, vision loss, limb amputation, cardiovascular events, and premature mortality (2). In response, there is a large literature on diabetes risk assessment and prediction.

Despite extensive research, current T2DM prediction, diagnosis, and prevention models continue to exhibit limitations. Most studies specify independent variables such as BMI, smoking status, or dietary habits and estimate their separate effects on incident T2DM, often using additive or risk-score based approaches (3). This design rarely captures interdependence, directionality, or the stability of links among diet, sleep, mood, activity, and demographics (4), and it frequently relies on downstream biomarkers such as HbA1c or fasting glucose that reflect established dysglycemia rather than upstream behavioral precursors (5). In this study, “signal” denotes a detectable pattern of interaction among behavioral variables, “marker” refers to an observable manifestation of this signal in the dataset, and “precursor” describes its appearance prior to deviation in conventional clinical biomarkers.

A study using UK Biobank shows that irregular sleep timing and duration are associated with higher risk of incident T2DM, even among individuals with adequate average sleep; however, sleep was analyzed as a single exposure and several measures were self reported (6). A dose-response meta analysis found that greater total and moderate to vigorous physical activity is linked to lower risk, often beyond the effect of adiposity, although activity was frequently self reported and evaluated in isolation (7). A classic cohort study reported that a composite lifestyle score combining dietary intake, physical activity, tobacco use, alcohol consumption, and body weight explained a large share of incident T2DM, but the score was additive by design and did not estimate directionality or stability among these behavioral domains (8).

Recent evidence further indicates that alcohol consumption contributes directly to vascular aging, a process closely linked to insulin resistance, impaired glucose delivery, and broader metabolic dysregulation that precede and contribute to T2DM development (9). Taken together, these studies demonstrate robust single-domain and additive effects of dietary intake, physical activity, tobacco use, alcohol consumption, and sleep behavior, but they do not show how relationships among diet, sleep, emotional states, physical activity, and demographic factors reorganize prior to diagnosis. Because T2DM is fundamentally a disorder of metabolic regulation, early risk may manifest not only through isolated behaviors but through loss of coordinated regulation across behavioral and psychosocial domains. In this study, the term behavioral coherence is used to denote the alignment and mutual reinforcement of emotional, dietary, sleep-related, physical activity, and social variables that support metabolic stability over time.

Many machine learning (ML) studies for incident T2DM use static baseline features and prioritize discrimination metrics (e.g., AUC), rather than modeling how behaviors interact; recent reviews of T2DM prediction consistently note this emphasis on performance over structure (10). Post-hoc explainers such as Shapley additive explanations (SHAP) (11) or partial dependence plots (PDPs) (12) add feature-wise attributions, but they do not recover inter-feature influence or its stability; moreover, PDPs can mislead under correlated predictors (13, 14), and SHAP explanations have documented pitfalls (13). Many models also include glycaemic and lipid biomarkers (e.g., HbA1c, fasting glucose, cholesterol), which improves accuracy but shifts modeling toward detection of established dysglycemia rather than preclinical behavioral disruption (15, 16). Broader evidence further shows that ML does not reliably outperform strong statistical baselines and needs better methodology and reporting in clinical prediction (17). This gap highlights the need for more integrative models that look at the whole system, capturing changes in everyday behaviors before diabetes or other metabolic diseases become clinically visible.

To address this gap, this study aims to develop and validate a behavioral framework for T2DM that detects early disruption in behavioral coherence and quantifies how links among sleep, mood, diet, physical activity, and demographics reorganize before clinical dysglycemia appears. We expected that, relative to healthy individuals, those who develop T2DM would exhibit a loss of stable, coherent relationships among these behavioral domains, with emotional and sleep-related states exerting more variable and destabilizing influences on dietary and lifestyle patterns. To test these expectations, a behavioral neural framework was developed and validated using longitudinal data from the UK Biobank.

### 1.1 Contributions of the study

This study makes four key contributions to the literature on T2DM risk prediction and behavioral modeling, advancing both methodological and conceptual understanding in the domain:

1. **Systems level behavioral risk framework:** This study introduces a dual-modeling framework that couples a Cox proportional hazards model with an ANN-based behavioral coherence and network analysis. This hybrid design enables both individual risk estimation and system-level mapping of interaction patterns among diet, emotion, sleep, and lifestyle variables, providing a holistic view of preclinical metabolic vulnerability within the cohort.
2. **Non-biochemical, behavior-centered early risk detection:** By excluding laboratory biomarkers and instead using longitudinal psychosocial, dietary, and lifestyle variables, the study demonstrates that accurate T2DM risk prediction is possible from everyday modifiable factors. This shift from biomarker-dependent models to a systems-level behavioral framework highlights how preclinical vulnerability can be detected through disruptions in sleep, mood, diet, and smoking patterns before dysglycemia becomes clinically measurable.
3. **Translating behavioral coherence from concept to measurable patterns:** This study advances behavioral coherence from a theoretical construct to a measurable, testable phenomenon. It identifies three reproducible signatures of disruption in T2DM: (i) emotional revaluation of foods, in which dietary items shift from stabilizing to strain-linked; (ii) erosion of demographic anchors, as age and BMI-defined weight status cease to structure behavior predictably; and (iii) instability in state-to-action mappings, reflecting fragmented and less reliable behavioral regulation. Together, these signatures provide an empirical basis for treating behavioral coherence as an early marker of metabolic vulnerability.
4. **From structural disruption to prevention targets:** By linking hazard ratios with structural roles in the behavioral network, the framework surfaces leverage points for intervention. Risk-amplifying behaviors such as smoking, processed meat intake, and irregular sleep emerge as destabilizing hubs, while stabilizing patterns such as high-fiber breakfasts and optimal sleep duration appear protective. Although not yet tested in interventions, these system-level priorities provide an evidence-informed foundation for designing psychologically attuned and personalized prevention strategies.

## 2 Related work

A substantial body of research has examined how clinical, behavioral, and lifestyle factors contribute to the incidence of T2DM (5, 17). Prior studies consistently demonstrate that risk is shaped not only by biological markers but also by everyday routines and psychosocial contexts (18, 19). Factors such as psychological distress and social connection, insomnia, sleep quality and circadian alignment, as well as dietary quality and broader pattern-level effects, have each been investigated in relation to T2DM onset (20–23). The following section reviews the literature to highlight how these domains have been studied, the consistent associations that have emerged, and the gaps that remain.

### 2.1 Psychological distress and social connection

Many studies have reported that psychological distress, encompassing both subclinical symptoms (e.g., persistent low mood, anxiety, sadness) and clinically diagnosed conditions (e.g., major depressive disorder), is associated with a higher incidence of T2DM. A prospective meta-analysis (24) found that individuals with depression or elevated depressive symptoms at baseline had a 60% higher risk of developing T2DM compared with those without depression, whereas having T2DM only modestly increased the risk of subsequent depression. Beyond clinical diagnoses, chronic psychosocial stressors in daily life have also been linked to elevated risk. An extensive European cohort study involving over 120,000 adults found that job strain was associated with a modest but significant increase in T2DM incidence, independent of lifestyle and demographic factors (25).

Beyond psychiatric symptoms, low or poor social connection, operationalized as loneliness or social isolation rather than social engagement, has emerged as an additional risk signal. For instance, multi-cohort and Mendelian randomization analyses link loneliness and social isolation to higher T2DM incidence across European and East Asian cohorts, with complementary evidence from national registry data (26, 27). Collectively, these studies establish psychological distress and weak social connection as meaningful contributors to T2DM risk. However, they tend to examine such factors independently or combine them into additive scores, overlooking how psychosocial states may dynamically interact with other behaviors such as diet or sleep in the lead up to diagnosis.

### 2.2 Sleep quality, insomnia, and circadian alignment

Prospective and meta analytic evidence indicates that insomnia symptoms including difficulty initiating or maintaining sleep and non restorative sleep are associated with higher incidence of T2DM, independent of common confounders (28). Primary care cohorts show that poor sleep quality elevates diabetes risk, with insomnia in prediabetes raising progression hazard by about 30% (29).

More recent device-based studies, using wrist-worn accelerometers and actigraphy-derived sleep metrics rather than glucose monitoring, demonstrate that irregular sleep timing and circadian misalignment predict incident T2DM even when total sleep duration is sufficient (30, 31). Experimental studies support these findings by showing that circadian disruption impairs glucose tolerance and insulin sensitivity, providing biological plausibility linking irregular sleep schedules, insomnia, and metabolic risk (32). In this study, sleep disturbance is operationalized specifically through self-reported insomnia symptoms, allowing us to evaluate its contribution to incident T2DM risk alongside diet and psychosocial factors and to test whether losses of behavioral coherence emerge when sleep regulation is disrupted.

### 2.3 Diet quality and eating patterns

Poor dietary intake contributes independent predictive value for T2DM. Researchers have found that higher consumption of red meat, particularly processed red meat, is associated with an increased risk of developing T2DM, a relationship confirmed across multiple U.S. cohorts and meta analyses (33). Conversely, higher whole grain intake is linked to lower risk (34), while Mediterranean like dietary patterns relate to reduced incidence and improved cardiometabolic profiles in both observational and intervention contexts (35). However, average main effects mask more nuanced processes. The emotional meaning of foods and the state dependent cues that drive choices may shift under psychosocial load or emerging illness. Moreover, timing of intake and its coupling with sleep patterns are often under measured, obscuring interactions between diet, circadian organization, and psychological state (36). Thus, while dietary risk is well established, its integration with other behavioral domains remains underexplored.

### 2.4 Integrative gap

Taken together, existing research demonstrates that adverse psychosocial states (e.g., psychological distress, low social connectedness), sleep disturbance, physical inactivity, and poor dietary quality are robustly associated with the incidence of T2DM. Yet most studies evaluate these variables independently, treat them additively in lifestyle scores, or rely heavily on downstream biomarkers. Such approaches clarify what the risk factors are, but not how their relationships reorganize, lose stability, or interact dynamically before clinical onset. The present study addresses this gap by moving from isolated exposures to a systems perspective, embedding these behavioral domains within a multi domain network. This enables us to estimate their incremental contribution to incident risk and to assess whether the relationships among psychosocial states, sleep, diet, and activity show shifts in direction or instability across health states, thereby operationalizing behavioral coherence as an early warning signal for T2DM.

## 3 Research methodology

**Figure 1:**
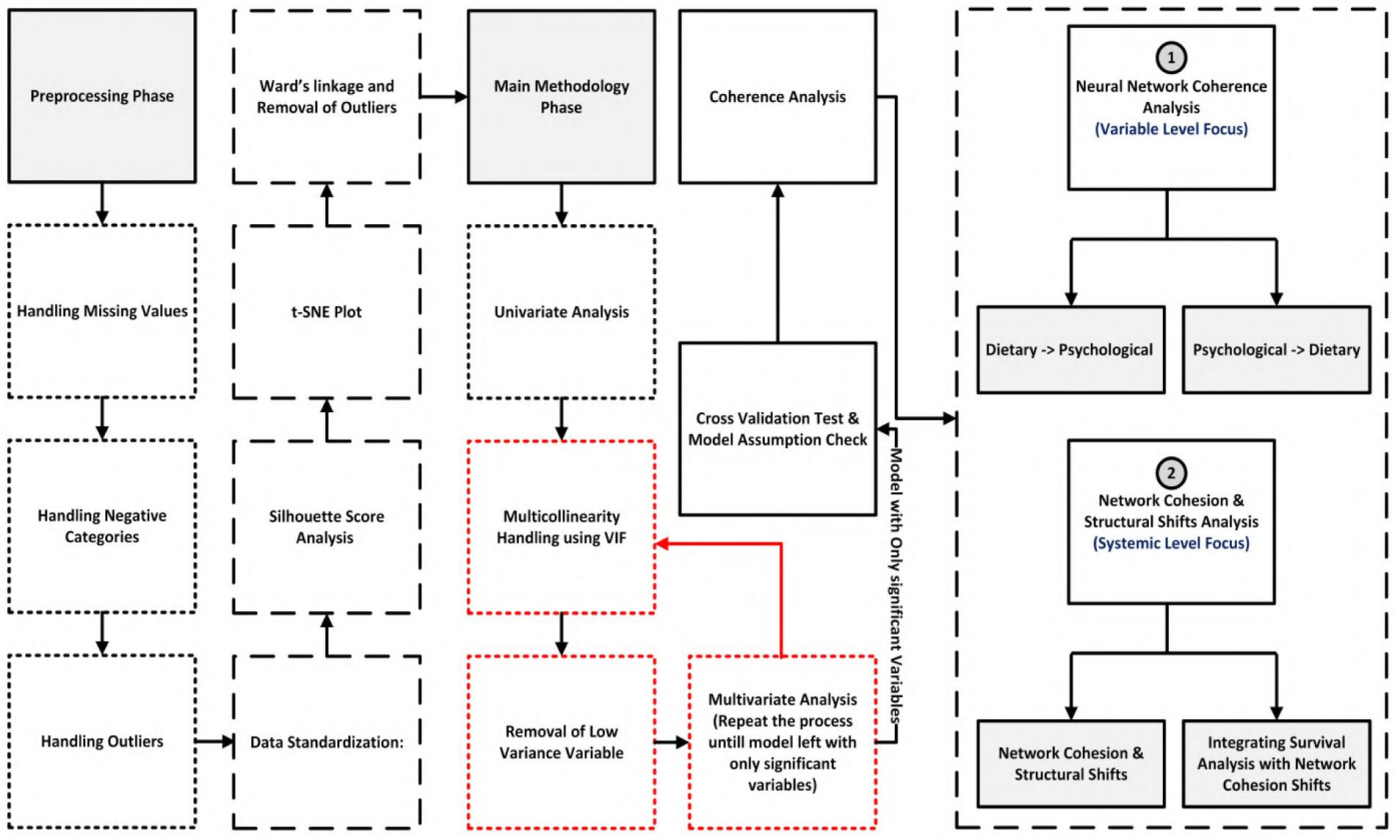
Overall architecture after data selection, outlining the analytic pipeline from data preprocessing to behavioral interaction mapping. The methodology is organized into four stages: (1) preprocessing with quality control, missing-data imputation, and normalization; (2) feature engineering and selection; (3) model training and validation with cross-validation; (4) neural network coherence analysis that maps influences between dietary and psychological variables at the variable level, and (5) system-level network cohesion and structural shift analysis that integrates these changes with the survival results.

This study employed a dual-modeling framework to investigate behavioral precursors of T2DM, integrating predictive risk estimation with systems-level analysis of behavioral coherence. Figure 1 shows the overall architecture after data selection, outlining the analytic pipeline from data preprocessing to behavioral interaction mapping. The methodology is organized into four main stages:

- **Dataset preparation:** UK Biobank data were filtered using strict inclusion criteria to support time-to-event analysis (Section 4).
- **Preprocessing and feature selection:** Standardization, imputation, and outlier exclusion were followed by univariate screening and multicollinearity checks (Section 5).
- **Survival modeling:** A multivariate Cox proportional hazards model (37) was used to analyze time to T2DM diagnosis with right censoring and irregular follow-up; its semi-parametric form leaves the baseline hazard unspecified and yields efficiently estimated, clinically interpretable hazard ratios. To ensure robustness, proportional hazards diagnostics and C-index discrimination were assessed, and model stability was evaluated via cross-validation (Sections 6–7).
- **Neural network analysis and cohesion shifts:** Behavioral interactions were modeled separately for diabetic and non-diabetic groups using a feedforward artificial neural network (ANN). Cohesion shifts were then quantified by comparing influence matrices and directional stability across health states (Sections 8–11).

This approach enabled both individual risk prediction and detection of systemic behavioral disintegration. Cohesion shifts provided a key signal of emerging metabolic vulnerability, capturing how emotional, psychosocial, and demographic anchors in behavior erode prior to clinical diagnosis.

## 4 Dataset preparation

**Figure 2:**
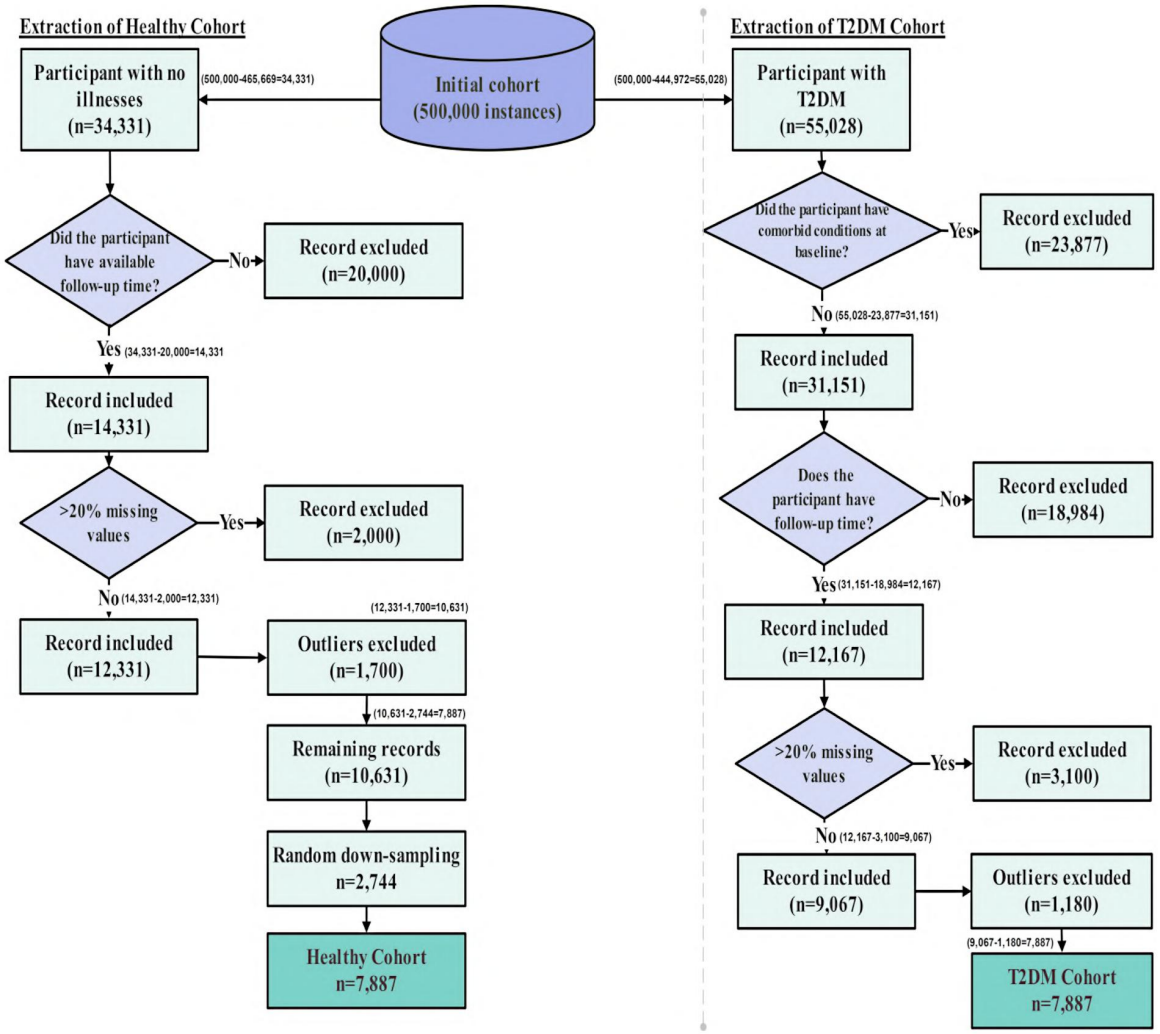
Flow diagram of participant selection from the UK Biobank cohort. Starting from approximately 500,000 records, inclusion and exclusion criteria were applied separately for T2DM and non-T2DM individuals. Records with comorbid illnesses, >20% missing data, and statistical outliers were excluded. Healthy individuals were randomly down-sampled to match the T2DM sample size. The final analytic dataset included 7,887 participants with T2DM and 7,887 healthy participants.

The dataset was derived from the UK Biobank (38), a prospective cohort of over 500,000 participants aged 40 to 69 at baseline. It included behavioral, psychosocial, demographic, and clinical variables such as validated measures of depression, insomnia, loneliness, self-reported mental health, physical activity, sleep, and diet, which were essential for modeling behavioral pathways and intervention outcomes. To construct the analytic dataset, we first separated participants into two parallel streams: (1) individuals with no record of T2DM, and (2) individuals with evidence of T2DM in linked health records. The full workflow is shown in Figure 2.

**Healthy stream:** The healthy cohort was defined using a strict baseline exclusion criterion. Participants were required to have no recorded chronic disease of any type, including T2DM. Disease status was determined using UK Biobank’s linked clinical records and self-reported medical history, with diagnoses identified via UK Biobank field **41270** (“Diagnoses ICD-10”). Individuals with any record of T2DM (ICD-10 code E11) or any other major chronic illness were excluded. Applying this criterion to the full UK Biobank cohort of approximately 500,000 participants resulted in the exclusion of 465,669 individuals. The remaining 34,331 participants were free of T2DM and all other major chronic diseases at baseline and were therefore designated as the healthy cohort. This group corresponds to the healthy branch shown in Figure 2, prior to follow-up verification, missing-data filtering, and outlier removal.

The rationale for this restriction was to isolate behavioral, dietary, and psychosocial patterns specifically attributable to diabetes rather than to general multimorbidity. Major non-diabetic chronic conditions, including cancer, chronic cardiovascular disease, chronic kidney disease, chronic respiratory disorders, and other long-term illnesses, are known to independently affect physical activity, diet, sleep behavior, and psychological well-being. Including such participants would confound diabetes-specific behavioral signatures with changes driven by unrelated disease processes. Restricting the healthy cohort to genuinely disease-free individuals therefore provided a clean reference group for identifying behavioral reorganization associated with the development of T2DM.

**T2DM stream:** T2DM was identified using ICD-10 codes E11.0–E11.9 recorded in UK Biobank linked hospital inpatient data, corresponding to Type 2 (non–insulin-dependent) diabetes mellitus. Specifically, these codes include diabetes mellitus with hyperosmolarity (E11.0), ketoacidosis (E11.1), renal complications (E11.2), ophthalmic complications (E11.3), neurological complications (E11.4), peripheral circulatory complications (E11.5), other specified complications (E11.6), multiple complications (E11.7), unspecified complications (E11.8), and diabetes mellitus without complications (E11.9). Using these diagnostic codes, we extracted all participants who had at least one recorded T2DM diagnosis in their linked medical history, resulting in 55,028 individuals. This step therefore excluded approximately 444,972 participants from the original UK Biobank cohort of 500,000 who had no recorded T2DM diagnosis. The figure of 55,028 reflects the initial size of the T2DM stream prior to comorbidity filtering, missing-data screening, or outlier removal, as illustrated in the right-hand branch of Figure 2.

T2DM frequently co-occurs with other long-term illnesses, including cancer, cardiovascular disease, chronic respiratory disorders, and inflammatory conditions, which can independently influence lifestyle behaviors and psychological outcomes. To isolate behavioral, dietary, and psychosocial patterns specifically associated with T2DM, individuals with major chronic comorbid conditions were excluded, thereby reducing confounding effects from non-diabetes–related illnesses. This approach enabled a more homogeneous T2DM cohort and improved interpretability of downstream behavioral analyses by limiting disease-related heterogeneity. After applying this comorbidity filter, 31,151 individuals remained in the T2DM stream, corresponding to the comorbidity-filtered cohort shown in Figure 2.

### 4.1 Follow-up time and eligibility for survival modeling

As shown in Figure 2, once the healthy and T2DM streams were established, the next step was to determine which participants had sufficient information to contribute a valid time-to-event interval. To ensure consistency in the survival analysis, both groups were then processed identically through follow-up verification, missing-data exclusion, and outlier removal.

- **Healthy participants (censored observations):** As the goal here was to analyze individuals who were healthy at baseline (UK Biobank field **53**) and remained free of T2DM throughout the observation window, follow-up time for these participants was defined from their baseline assessment to the most recent subsequent assessment at which they were still recorded as non-diabetic. Many participants attended a second or third assessment visit; in such cases, the latest visit confirming continued non-diabetic status was used to determine the censoring time. Participants who had no follow-up assessment and no later linked hospital records could not provide a verifiable time interval during which they were known to remain healthy, and were therefore excluded. After applying this criterion, 14,331 healthy participants had valid follow-up time.
- **Participants who developed T2DM (event observations):** As outlined above, incident T2DM was identified using ICD-10 codes E11.1-E11.9. These diagnoses are captured in UK Biobank’s linked Hospital Episode Statistics dataset, where field “130708” records the exact date on which an E11 diagnosis was first documented. For each participant, follow-up time was calculated as the interval between the baseline assessment and this first diagnosis date provided the diagnosis occurred after baseline. This method produces valid survival times even for individuals who never returned for a second assessment visit, because their diagnosis date is captured through hospital linkage. After applying this rule, 12,167 T2DM participants had a valid event time.

This approach ensures that all individuals included in the survival model have a clearly defined period during which they were at risk, thereby preventing misclassification of event or censoring times and improving the accuracy of the hazard estimates. After establishing valid follow-up times, we next applied the missing-data and outlier exclusion criteria to the 14,331 healthy records and 12,167 T2DM records retained at this stage.

## 5 Data preprocessing and feature selection

To ensure data integrity and analytical robustness, several preprocessing steps were undertaken. Clinical biomarker variables (e.g., glucose, HbA1c, and cholesterol) were intentionally removed at the outset to focus the analysis on modifiable, non-clinical behavioral and psychosocial predictors. Although glycaemic and lipid biomarkers are clinically actionable and may be intervened upon at sub-clinical levels (5), their inclusion would shift the modeling task toward detection of emerging dysglycemia rather than the identification of earlier behavioral system disruption. The aim of this study was therefore not to replace biomarker-based risk assessment, but to examine whether vulnerability to T2DM can be detected through changes in everyday behavioral regulation prior to laboratory abnormalities becoming apparent.

Following this conceptual scoping of predictors, standard data-quality preprocessing procedures were applied to prepare the analytic dataset. Records (rows) with more than 20% missing data were excluded. This threshold reflects common pragmatic practice in epidemiological and machine-learning analyses to balance data completeness with sample retention, and to limit bias, instability, and loss of efficiency associated with extensive missingness and unreliable imputation (39–41). Applying this threshold to the 14,331 healthy participants with valid follow-up removed 2,000 records, leaving 12,331 healthy individuals. Likewise, from the 12,167 participants with incident T2DM, 3,100 records exceeded the missing-data threshold and were removed, resulting in 9,067 T2DM participants.

For the remaining missing values, categorical responses such as “Don’t know” and “Prefer not to answer” were coded as Not a Number (NaN) and imputed using mode imputation for categorical variables, mean imputation for continuous variables, and the least frequent category for binary fields. Negatively coded responses (e.g., −7 for “None of the above”) were recoded to maintain logical ordering.

### 5.1 Outlier handling and detection

**Figure 3:**
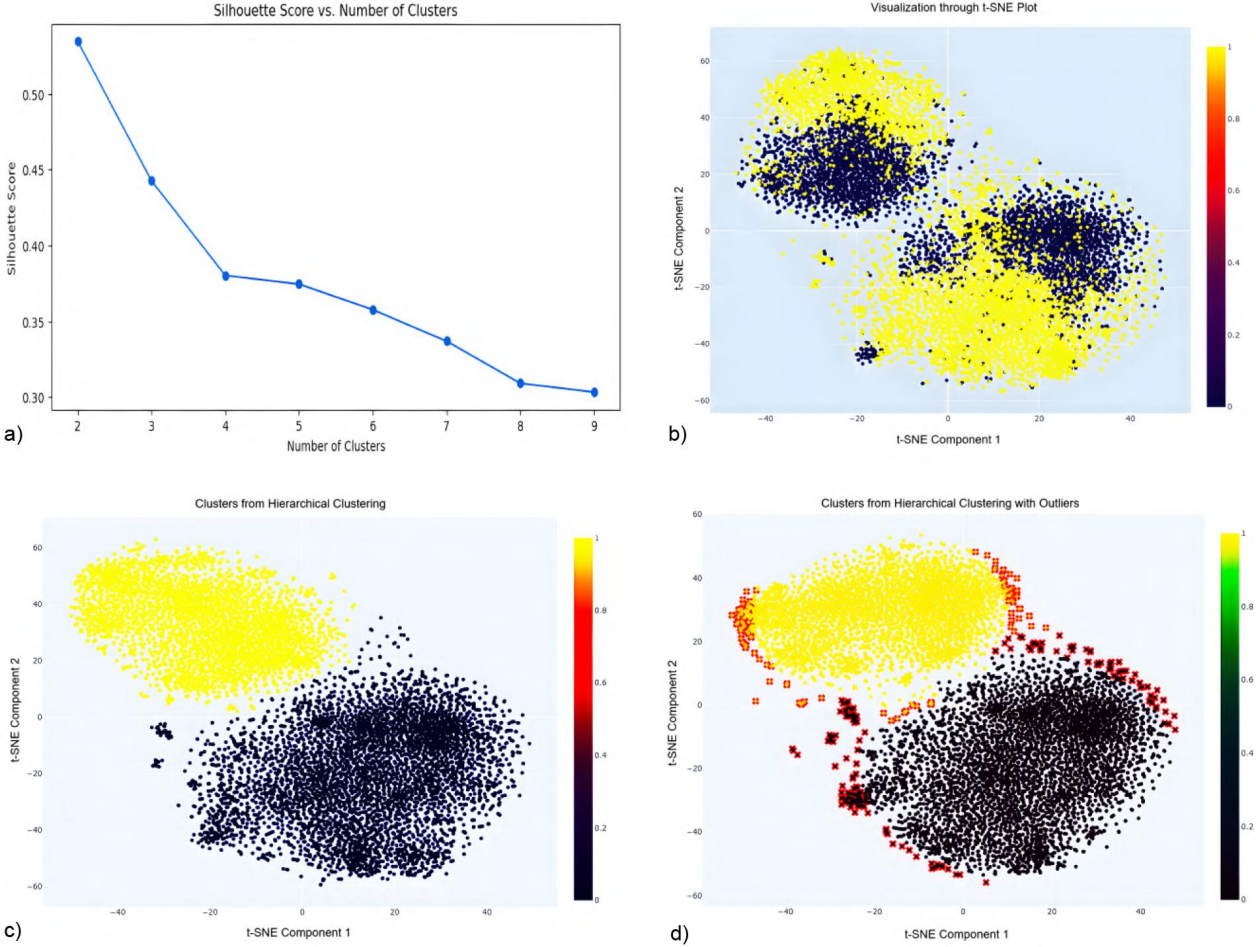
Silhouette score analysis **(a)** indicates optimal clustering at *k*=2. The *t*-SNE projection **(b)** visually separates these clusters, highlighting outliers at the data margins. Hierarchical clustering using Ward’s linkage **(c)** refines cluster integrity, while final visualization **(d)** reveals peripheral outliers (red) subsequently removed to enhance data quality and improve model robustness. **(a)** Silhouette score vs. No. of clusters. **(b)** Visualization through *t*-SNE Plot. **(c)** Clusters from hierarchical clustering. **(d)** Clusters with outliers highlighted.

Following missing-data filtering and imputation applied only to records with limited missingness (<20%), outlier handling was performed to address structurally inconsistent behavioral profiles that could distort multivariate relationships and bias survival estimates. Unlike missingness, which was addressed through targeted value-level imputation when limited, outliers were handled using a full-case removal strategy, as adjusting extreme values can disrupt inter-variable dependencies and obscure system-level structure. Entire records identified as outliers were therefore removed to preserve multivariate integrity and ensure stable estimation in subsequent Cox proportional hazards models. Outlier detection was performed prior to supervised modeling to avoid data leakage. Figure 3 illustrates the full outlier detection workflow, from optimal cluster selection to identification of peripheral observations removed from the dataset.

#### 5.1.1 Outlier detection pipeline

A multi-step unsupervised procedure was applied to identify and eliminate structurally inconsistent data points, creating a stable dataset for analysis while maintaining a balance between generalizability and internal validity.

- **Data standardization:** All features were standardized to a mean of zero and standard deviation of one to ensure equal contribution, especially for algorithms like t-distributed Stochastic Neighbor Embedding (*t*-SNE) (42), which are sensitive to feature scale.
- **Optimal cluster selection via Silhouette score:** Silhouette analysis (43) was used to evaluate clustering quality, with the highest score at k=2, indicating optimal cluster separation (see Figure 3a).
- ***t*-SNE visualization:** *t*-SNE was applied to project the dataset into two dimensions using a perplexity of 40 and 5,000 iterations. It was chosen over principal component analysis (PCA) (44) because it more effectively preserved local neighborhood structures in the data used in this study, which facilitated clearer cluster separation and outlier detection. The resulting two-dimensional projection revealed two distinct clusters corresponding to the binary target variable, along with peripheral points that may represent outliers (see Figure 3b).
- **Hierarchical clustering:** Ward’s linkage method (45) was used on the *t*-SNE components, producing well-separated clusters with minimized intra-cluster variance (see Figure 3c).
- **Outlier identification and removal:** Outliers were identified based on their Euclidean distance from the centroid of their respective clusters:
  - ***Centroid calculation:*** Centroids of each cluster in *t*-SNE space were computed.
  - ***Distance measurement:*** Euclidean distances between each data point and its cluster centroid were calculated.
  - ***Thresholding:*** Points beyond the 95th percentile of these distances were flagged as outliers.
  - ***Visualization and removal:*** Outliers were marked in red in Figure 3d and subsequently removed to avoid skewing the analysis.

This systematic pipeline ensured the removal of structurally inconsistent data points, resulting in improved cluster cohesion and enhanced analytical integrity. Although this approach strengthens internal validity and reduces model bias, it necessarily involves a trade-off in external generalizability, as the removal of extreme cases may reduce representation of certain subpopulations. Nonetheless, for time-to-event modeling and simulation purposes, this trade-off was deemed acceptable to support stable and unbiased survival estimation. Applying the pipeline to the UK Biobank cohorts removed an additional 1,700 outliers from the healthy cohort and 1,180 from the T2DM cohort, reducing the samples from 12,331 to 10,631 and from 9,067 to 7,887 participants, respectively, forming the pre-balanced cohorts used in the subsequent survival analysis.

### 5.2 Final analytic cohort derivation

After establishing valid follow-up times and completing the missing-data and outlier filtering steps described above, the resulting healthy and T2DM cohorts were carried forward to the final stage of cohort construction. Because the healthy pool remained substantially larger than the T2DM cohort after preprocessing (10,631 vs. 7,887), random down-sampling was applied to the healthy group to achieve matched sample sizes. Sampling was performed without conditioning on any predictor variables, ensuring that the procedure was independent of demographic, dietary, psychological, and lifestyle characteristics. Although this step alters the observed prevalence of T2DM relative to the full UK Biobank population, random selection preserves marginal structure in expectation. This balanced dataset was used exclusively for model training rather than for estimating population-level prevalence. Because Cox proportional hazards estimation relies on time-to-event information through the partial likelihood rather than outcome prevalence, altering class proportions through random down-sampling does not bias hazard ratio estimation or survival time interpretation (46–48).

As shown in Figure 2, the final analytic dataset consisted of two equal groups of 7,887 participants each: one comprising individuals who were healthy at baseline but later developed T2DM during follow-up (T2DM cohort), and one comprising individuals who maintained a non-diabetic status across all available observations (Healthy cohort). Time-to-event durations ranged from 1 to 17 years, providing sufficient longitudinal variability for stable and unbiased survival estimation.

## 6 Survival modeling and risk identification

This section outlines the Cox modeling pipeline, covering variable screening, multicollinearity control, proportional hazards testing, and model validation, followed by interpretation of significant predictors forming the basis for simulation and intervention in the DT system.

### 6.1 Cox model development and validation

A penalized Cox proportional hazards model (37) was employed to estimate T2DM onset risk. The model simultaneously identified significant predictors and generated time-sensitive risk estimates, enabling personalized simulation. The hazard function is given in Equation 1:

$$h(t|X)=h_{0}(t)\cdot \exp(\beta_{1}X_{1}+\beta_{2}X_{2}+\cdots +\beta_{p}X_{p})$$ (1)

where h(t∣X) is the hazard at time t, $h_{0}(t)$ is the baseline hazard, and $\exp(\beta_{i})$ represents the hazard ratio (HR) for predictor $X_{i}$.

#### 6.1.1 Univariate analysis

To identify predictors of diabetes onset, univariate Cox proportional hazards regression was performed for each variable, modeling time to T2DM onset as a function of a single predictor. Statistical significance was assessed using *p-values*, with variables meeting the threshold of p<0.05 retained for multivariate analysis. Categorical predictors were transformed into dummy variables to enable inclusion in the Cox framework while minimizing multicollinearity. It should be noted that univariate screening was used solely as an initial dimensionality reduction step and not as a criterion for final variable selection, which was determined in the multivariate modeling stage.

To improve stability and interpretability of the final model, two quality control procedures were applied. First, dummy variables with low variance across the dataset or within subgroups stratified by event status were excluded to prevent unstable coefficient estimates. Second, multicollinearity was addressed using the Variance Inflation Factor (VIF), defined as $\text{VIF}=1/(1-R_{j}^{2})$, where $R_{j}^{2}$ is obtained by regressing predictor $X_{j}$ on all other predictors. Variables with VIF>10 were removed, consistent with established guidelines (49).

From 90 features evaluated, 27 were initially significant in univariate analysis. Following variance and multicollinearity screening, only robust, independent, and reliable predictors were advanced to the multivariate Cox model, ensuring improved validity and generalizability of results.

#### 6.1.2 Multivariate analysis

After univariate screening, all candidate predictors were entered simultaneously into an initial multivariate Cox proportional hazards model to evaluate their joint effects on time to T2DM onset. This approach explicitly accounts for interdependencies among predictors and allows effect estimates to change in the presence of other variables. The 27 features identified in univariate analysis were included in the initial model, and several predictors lost significance once confounding and shared variance were accounted for. Variable retention was determined using an iterative stepwise procedure, with predictors removed only when they contributed no independent explanatory value after joint adjustment and exhibited redundancy or instability.

Because removing correlated predictors can alter estimated coefficients, statistical significance alone was not treated as sufficient justification for exclusion. Variables were removed only when they contributed no incremental explanatory value in the multivariable context and exhibited redundancy or instability due to shared variance (supported by VIF screening) or low-variance categories yielding unreliable estimates. This process reduced the predictor set from 27 to 18, underscoring the importance of joint modeling rather than variable-by-variable selection. As a sensitivity check, models were refit retaining theoretically supported covariates, and the direction and magnitude of key predictors as well as the overall conclusions remained unchanged. The final reduced predictor set was then used consistently in both the Cox survival model and the ANN analysis to ensure comparability between individual risk estimation and system-level interaction modeling.

The coefficients ($\exp(\beta_{i})$) represent adjusted hazard ratios, quantifying the relative risk of T2DM onset associated with each predictor while holding other factors constant. For instance, a dietary factor with a hazard ratio greater than 1 indicates an increased risk of T2DM independent of demographic or lifestyle covariates. This stepwise refinement enhanced both interpretability and robustness, yielding a final model focused on key predictors that reliably contribute to T2DM risk.

#### 6.1.3 Proportional hazards assumption

Following multivariate modeling, the proportional hazards assumption was evaluated to ensure the validity of the Cox regression framework. This assumption requires that the effect of each covariate on the hazard remains constant over time, implying that hazard ratios are time-invariant. For example, if higher physical activity reduces diabetes risk by 30% (hazard ratio=0.70), this relative effect is expected to persist throughout the follow-up period.

The proportional hazard assumption was assessed using diagnostic methods based on Schoenfeld residuals (50). Violations can bias hazard ratio estimates; therefore, corrective strategies were applied when necessary. Two approaches were implemented: (i) stratification, allowing the baseline hazard to vary across covariate strata, and (ii) incorporation of time-dependent covariates to capture changing effects over time. In this study, non-proportionality was detected for cooked vegetable intake and long-standing illness, both of which were modeled with covariate–time interactions to preserve validity and interpretability. These adjustments enabled the model to accommodate dynamic predictor effects, thereby enhancing explanatory accuracy while preserving the validity of the proportional hazard assumption.

#### 6.1.4 Internal validation

Model performance was evaluated using 10-fold stratified cross-validation, training on nine folds and testing on the tenth (51). Stratification preserved the distribution of diabetes onset and censored cases across folds. L2 regularization reduced overfitting by penalizing large coefficients (52). Performance was measured with the concordance index (C-index) (53), yielding a mean of 0.90 (SD=0.004), indicating excellent accuracy and consistency. The model was then refitted on the full dataset to maximize information, and the proportional hazards assumption was re-verified, confirming the stability and reliability of the final model.

## 7 Results and interpretation of significant predictors

This section presents the findings from the multivariate Cox proportional hazards model, emphasizing their implications for disease prediction, behavioral interventions, and dietary profiling in T2DM risk. The model highlights the interplay of psychosocial stressors, sleep patterns, dietary choices, and demographic factors in shaping T2DM risk. Several modifiable, non-clinical variables, including mental well-being, sleep behavior, and specific dietary components, emerged as significant predictors. Table 1 summarizes the selected predictors along with their coefficients (β), hazard ratios (exp⁡(β)), percentage change in hazard (HR%), confidence intervals, and *p*-values. HR% was calculated as exp⁡(β)−1)×100, and values are rounded consistently.

**Table 1:** Final selected variables and corresponding hazard ratios for T2DM risk. Reference categories are detailed in the table note.

Risk factors	β	exp⁡(β)	HR (%)	*p*	95% CI for exp⁡(β)	
					Lower	Upper
Loneliness isolation	0.13	1.14	14.00	p<0.001	1.07	1.21
Seen a psychiatrist	0.16	1.18	18.00	p<0.001	1.09	1.27
Sleeplessness insomnia	0.06	1.06	6.00	0.02	1.00	1.12
Fed up feelings	0.08	1.08	8.00	p<0.001	1.02	1.14
Tense highly strung	0.15	1.17	17.00	p<0.001	1.10	1.25
Sleep duration 7–8 h	−0.13	0.87	−13.00	p<0.001	0.82	0.93
Nap during day	0.09	1.10	10.00	p<0.001	1.01	1.20
Difficulty getting up in morning	0.04	1.04	4.00	p<0.001	1.01	1.08
Plays computer games	0.04	1.04	4.00	p<0.01	1.01	1.07
Smoking status (Previous smokers)	0.12	1.13	13.00	p<0.001	1.07	1.19
Smoking status (Current smokers)	0.17	1.19	19.00	0.01	1.02	1.38
Current tobacco smoking	0.29	1.34	34.00	p<0.001	1.14	1.58
Salt added to food	0.04	1.05	5.00	p<0.001	1.02	1.08
Cheese intake	−0.05	0.94	−6.00	p<0.001	0.92	0.96
Brown bread	0.16	1.18	18.00	p<0.001	1.09	1.27
White bread	0.26	1.30	30.00	p<0.001	1.18	1.43
Other type of bread	0.11	1.11	11.00	0.07	0.98	1.26
Processed meat intake (Less than once a week)	0.07	1.07	7.00	0.15	0.97	1.19
Processed meat intake (Once a week)	0.05	1.05	5.00	0.29	0.95	1.17
Processed meat intake (2–4 times a week)	0.06	1.06	6.00	0.21	0.96	1.18
Processed meat intake (5–6 times a week)	0.17	1.18	18.00	0.03	1.01	1.38
Beef intake	0.16	1.18	18.00	p<0.001	1.08	1.29
Biscuit cereal	0.10	1.11	11.00	0.01	1.02	1.21
Oat cereal	−0.23	0.79	−21.00	p<0.001	0.73	0.85
Muesli	−0.55	0.57	−43.00	p<0.001	0.52	0.63
Sugary cereal	0.10	1.11	11.00	0.01	1.02	1.21
Age_[50–59]	−0.06	0.93	−7.00	0.50	0.75	1.14
Age_[60–70]	0.42	1.52	52.00	p<0.001	1.24	1.87
BMI_[25–29.9]_Overweight	−0.71	0.49	−51.00	p<0.001	0.39	0.60
BMI_[30–34.9]_Obesity class I	0.08	1.08	8.00	0.40	0.88	1.33
BMI_[35–39.9]_Obesity class II	0.43	1.54	54.00	p<0.001	1.26	1.89
BMI_[40-50]_Obesity class III	0.58	1.79	79.00	p<0.001	1.45	2.20

Psychosocial, sleep-related, and behavioral predictors were modeled as binary variables. *Salt added to food* was treated as an ordinal variable. *Processed meat intake*, *beef intake*, *bread type*, *cereal type*, *age*, and *BMI* were modeled as categorical variables using dummy coding. Reference categories were: Processed meat intake=never; Beef intake=never; Bread type=wholewheat bread; Cereal type=bran cereal; Age=40–49 years; BMI=18.5–24.9 kg/$m^{2}$ (normal weight).

Although several candidate variables have strong theoretical and empirical links to T2DM risk, such as alcohol consumption and physical activity (9, 54, 55), inclusion in the final model reflects their independent contribution in the multivariate context rather than on prior evidence alone. As described above, all candidate predictors were initially screened and evaluated jointly in multivariate modeling. Variables were excluded from the final model if they showed no independent association after adjustment, exhibited redundancy due to shared variance, had low variance or sparse response distributions leading to unstable estimates, or exceeded predefined preprocessing thresholds. For example, alcohol consumption and physical activity-related variables were evaluated but did not retain independent explanatory value after adjustment for correlated behavioral and psychosocial factors and were therefore not included in Table 1. This approach prioritizes model stability and interpretability while acknowledging the broader theoretical relevance of excluded factors.

To ensure transparency in variable interpretation, all predictors were coded in accordance with their native UK Biobank structures using binary or dummy-coded categorical variables to maintain interpretability and consistency within the Cox regression framework. Psychosocial, sleep-related, and behavioral exposures (e.g., loneliness, daytime napping, insomnia) were treated as binary indicators. Dietary variables with multiple intake-frequency levels were represented through mutually exclusive dummy variables, with the lowest-consumption category serving as the reference.

Specifically, “Salt added to food” is an ordinal variable with four levels (never/rarely, sometimes, usually, always). In the multivariate Cox model, these levels exhibited highly similar effect sizes and several were non-significant; therefore, the overall ordinal effect was retained and reported as a single hazard ratio. In contrast, processed meat intake comprises six ordered categories (never, less than once per week, once per week, 2–4 times per week, 5–6 times per week, daily) and these levels demonstrated distinct risk gradients. All categories were included as dummy variables with “never” as the reference, although only the 5–6/week category remained significant in the final model.

Beef intake is recorded on the same frequency scale, but only the highest consumption level (≥5/week or daily) retained statistical significance following stepwise model refinement and is therefore presented with “never” as the reference. Body mass index (BMI) was modeled using WHO-standard categories. The underweight category (<18.5 kg/$m^{2}$) contained too few participants for stable estimation and was therefore excluded; normal weight (18.5–24.9 kg/$m^{2}$) served as the reference category for all BMI contrasts. Age on the other hand was modeled using UK Biobank decadal categories (40–49, 50–59, 60–70 years), with 40–49 years defined as the reference group; this approach captures non-linear age-related risk gradients without imposing parametric assumptions.

This coding strategy preserves fidelity to UK Biobank variable structure while ensuring that only categories contributing independent explanatory value are retained in the multivariate model.

### 7.1 Psychosocial stressors and sleep behavior

Psychological strain was consistently associated with elevated T2DM hazard. Loneliness or social isolation, psychiatric consultation, and emotional distress were each associated with a 14%–18% increase in hazard, while frequent feelings of being fed up were associated with an 8% increase. Sleep-related factors also contributed independently. Insomnia symptoms and difficulty waking were associated with 6% and 4% higher hazard, respectively, and daytime napping with a 10% increase. In contrast, habitual sleep duration of 7–8 h per night was associated with a 13% lower hazard, indicating a protective association of adequate sleep duration.

Importantly, this protective association with sleep duration was observed alongside indicators of sleep disturbance and psychosocial stress, suggesting that aspects of sleep regulation beyond duration may be relevant for metabolic risk. Consistent with prior evidence, psychosocial stress and circadian disruption have been shown to impair glucose regulation through neuroendocrine and inflammatory pathways, underscoring the relevance of behavioral and chronobiology-informed interventions (56).

### 7.2 Dietary habits and composition

Dietary patterns showed strong yet heterogeneous associations with T2DM. High beef intake (≥5 servings/week) was associated with an 18% higher hazard, consistent with previous links between red meat and metabolic dysfunction (57). Processed meat intake showed mixed results: only individuals consuming processed meats 5–6 times per week had a significantly higher hazard (18%), while lower intake frequencies were not statistically significant. Adding salt to food, measured as an ordinal self-reported behavior (never/rarely to always), was associated with a modest 5% increase in hazard. In contrast, *cheese intake* was associated with a 6% lower hazard, possibly due to vitamin K2 and other bioactive components in fermented dairy that improve insulin sensitivity and glucose regulation (58).

These dietary variables represent key modifiable factors and serve as critical leverage points for targeted dietary interventions. Grain-based foods revealed notable contrasts. Brown bread was associated with an 18% higher hazard compared with the reference category of wholewheat bread, suggesting that not all breads confer equal benefits. Notably, white bread showed a stronger association with elevated risk, with approximately a 30% higher hazard relative to wholewheat bread. This pattern suggests that consumption of more refined grain products may be associated with higher T2DM risk within this cohort, consistent with prior prospective and meta-analytic evidence (54). Cereal types also diverged: muesli and oats reduced hazard by 43% and 21%, respectively, whereas sugary cereals and biscuit-type cereals increased hazard by 11%. These findings emphasize that foods within the same category may exert opposite metabolic effects depending on type and processing (59).

### 7.3 Smoking behavior

Smoking demonstrated a clear gradient of hazard compared with never smokers: Former smokers had a 13% higher hazard, current smokers 19%, and individuals actively smoking tobacco at baseline 34%. These findings are consistent with prior evidence that both historical and current exposure exert lasting metabolic burdens (60). In this study, smoking status captured lifetime history (never, previous, current), while current tobacco smoking reflected active use at baseline. Their joint inclusion highlights that the long-term metabolic hazards from past smoking differ from the acute hazards of ongoing exposure, enabling models to better distinguish historical vs. modifiable behaviors and refine predictions of individual hazard trajectories and intervention timing.

### 7.4 Age and BMI

Age showed a clear gradient in T2DM hazard. Adults aged 60–70 years had a 52% higher hazard compared with the reference group of 40–49 years, while those aged 50–59 years showed a non-significant 7% lower hazard. Body mass index (BMI), categorized according to WHO classification (61), demonstrated heterogeneous associations with T2DM risk. Overweight individuals (BMI 25–29.9 kg/$m^{2}$) exhibited a 51% lower hazard compared with the normal-weight reference group, a counterintuitive pattern sometimes described as the “metabolically healthy overweight” phenomenon (62). Importantly, BMI reflects body mass relative to height and does not directly measure adiposity or body fat distribution; therefore, these findings should not be interpreted as evidence of lower adiposity among individuals classified as overweight. The apparent protective association may reflect survivor bias, reverse causation, residual confounding, or differences in fat distribution and metabolic profile that are not captured by BMI alone (63). In contrast, obesity class I (BMI 30–34.9 kg/$m^{2}$) was not significantly associated with T2DM hazard, whereas obesity class II (BMI 35–39.9 kg/$m^{2}$) and class III (BMI 40–50 kg/$m^{2}$) were associated with substantially higher hazards of 54% and 79%, respectively. These results highlight the increasing metabolic risk associated with higher BMI categories, particularly at more severe levels of obesity.

To summarize, the Cox model identifies psychosocial stress, sleep behavior, diet, smoking, and BMI as key determinants of T2DM hazard. Modifiable lifestyle factors, including mental well-being, sleep quality, and dietary composition, emerged as critical intervention targets. The findings also highlight heterogeneity within BMI categories, with obesity driving substantial increases in hazard while overweight status shows more complex effects, reflecting the importance of fat distribution and metabolic health. Overall, prevention efforts should adopt an integrated approach that considers psychological, behavioral, and physiological factors to more effectively reduce T2DM hazard.

### 7.5 Risk score derivation and group stratification

Following internal validation of the multivariate Cox model, individual risk scores were computed to quantify each participant’s relative susceptibility to T2DM onset. The risk score for participant i was defined as the model’s linear predictor as shown in Equation 2:

$${\text{Risk Score}}_{i}=\sum_{\;j=1}^{\;p}\beta_{j}X_{ij}$$ (2)

where $\beta_{j}$ are the coefficients estimated from the final multivariate Cox proportional hazards model (Table 1), and $X_{ij}$ represents the participant-specific value for predictor j. Categorical predictors were represented using dummy variables, with reference categories specified in Table 1. The resulting score provides an interpretable log-relative hazard reflecting the combined contributions of behavioral, psychosocial, sleep-related, dietary, and demographic factors.

Because the linear predictor is continuous, a supervised discretization procedure was applied to generate clinically interpretable strata. A decision tree classifier (64) was trained using the risk score as the sole input and observed T2DM incidence as the target outcome. To balance interpretability and discriminatory performance, the tree was constrained to five leaf nodes and trained using 10-fold cross-validation, with the resulting split points fixed and applied uniformly to all participants. These thresholds defined the score ranges for the very low (−1.67 to 0.22), low (0.23 to 0.40), moderate (0.41 to 0.70), high (0.71 to 1.11), and very high (1.12 to 2.75) risk strata.

For transparency, two illustrative calculations demonstrate how the linear predictor maps to the final risk strata. Consider an individual who reports loneliness $\left(X_{1}=1\right)$, a habitual sleep duration of 7–8 h $\left(X_{2}=1\right)$, and regular muesli consumption $\left(X_{3}=1\right)$, with all remaining predictors fixed at their reference levels. The corresponding linear predictor is therefore 0.13−0.13−0.55=−0.55, which falls within the *Very Low* risk category. As a contrasting example, an individual exhibiting loneliness $\left(X_{1}=1\right)$, daytime napping $\left(X_{2}=1\right)$, current smoking $\left(X_{3}=1\right)$, sugary cereal intake $\left(X_{4}=1\right)$, and membership in the 60–70 year age group $\left(X_{5}=1\right)$ yields a linear predictor of 0.13+0.09+0.17+0.10+0.42=0.91, corresponding to the *High* risk category. These examples illustrate how specific combinations of behavioral and demographic variables translate into distinct diabetes risk classifications.

### 7.6 Validation of risk groups

**Figure 4:**
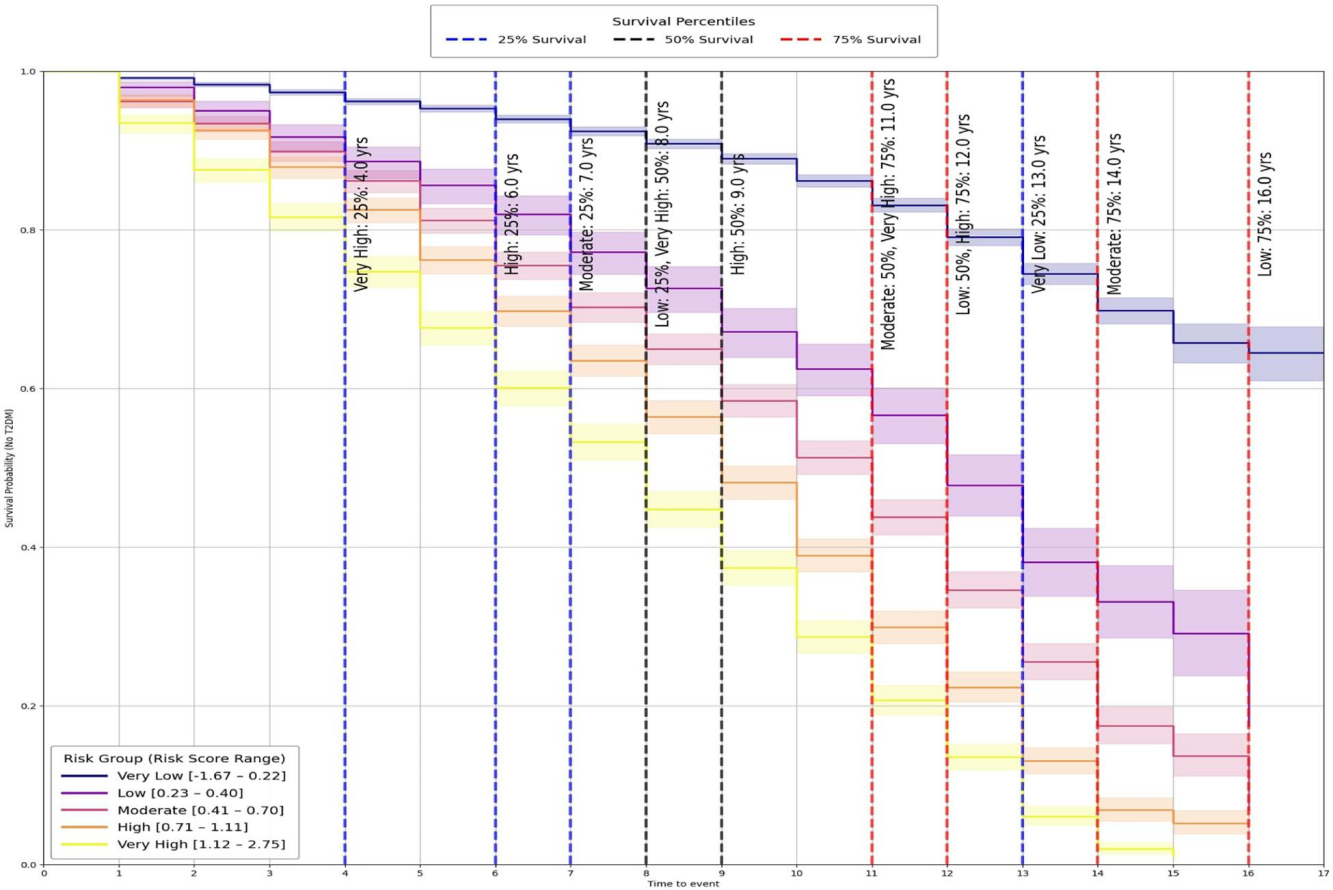
Kaplan-Meier survival curves by risk group.

The predictive utility of these groups was evaluated using Kaplan–Meier (KM) survival analysis (65), which revealed clear, progressive separation across categories (Figure 4). Pairwise log-rank tests (66) further confirmed that differences between groups were statistically significant (p<0.001, Table 2a), demonstrating that the stratification captures both major and incremental variations in diabetes onset risk.

**Table 2:** Panel **(a)** reports pairwise log-rank test outcomes: *Comparison* shows the two groups tested, *Test Statistic* is the log-rank $\chi^{2}$ value, and *p-value* indicates significance. Panel **(b)** shows survival time percentiles for each group, with *Score Range* defining the risk category and *25%*, *50%* (median), and *75%* giving survival times in years.

(a) Pairwise log-rank test results				
Comparison	Test statistic		*p*-value	
Very low vs very high	5255.49		p<0.001	
Very low vs high	3662.31		p<0.001	
Very low vs moderate	2072.38		p<0.001	
Low vs very high	409.51		p<0.001	
Moderate vs very high	353.93		p<0.001	
Very low vs low	517.59		p<0.001	
Low vs high	189.67		p<0.001	
Moderate vs high	95.38		p<0.001	
High vs very high	89.86		p<0.001	
Low vs moderate	46.71		p<0.001	
(b) Survival time percentiles by risk group				
Risk group	Score range	25%	50%	75%
Very low	[−1.67, 0.22]	13.00	∞	∞
Low	[0.23, 0.40]	8.00	12.00	16.00
Moderate	[0.41, 0.70]	7.00	11.00	14.00
High	[0.71, 1.11]	6.00	9.00	12.00
Very high	[1.12, 2.75]	4.00	8.00	11.00

“∞” denotes that survival did not fall below the percentile threshold during follow-up.

To provide clinically interpretable timelines, survival percentiles were also computed (Table 2b). Individuals in the *Very High* group reached 25% incidence by year 4 and 75% by year 11, whereas the *Very Low* group crossed the 25% threshold only after 13 years and did not reach higher incidence levels during follow-up.

Taken together, this stratification highlights the practical utility of the digital twin framework beyond statistical validation. By translating continuous outputs from the Cox model into discrete, time-sensitive categories, the system provides thresholds that can inform clinical decision-making and public health planning. These findings show that psychosocial and behavioral risk factors can be structured into groups that are both statistically distinct and clinically meaningful, reinforcing the value of this framework for individualized prevention and population-level screening.

## 8 From individual risk to system-level dynamics

While the Cox model identifies which individual variables increase or reduce the hazard of T2DM, it does not reveal how these same variables interact with one another or how broader behavioral–dietary systems reorganize as the disease develops. Many risk factors do not operate in isolation but influence and reinforce each other through emotional, lifestyle, and dietary pathways (67, 68). To understand not only who is at greater risk but also how behavioral regulation changes once T2DM emerges, we therefore extend the analysis using an ANN-based coherence framework. This approach complements the Cox model by mapping variable-to-variable influence patterns, quantifying structural shifts in behavioral cohesion, and identifying which predictors function as network hubs, bridges, or feedback drivers in the chronic-disease state. In this way, the ANN analysis adds a systems perspective to the survival model by linking hazard ratios to each variable’s systemic role within the broader behavioral-emotional-dietary network.

The remainder of this section outlines the ANN architecture, the preprocessing steps applied to ensure comparability with the Cox model, and the methodological procedures used to derive and interpret influence matrices. This approach prepares the groundwork for the network cohesion and interaction mapping discussed later in the manuscript.

### 8.1 Neural network architecture

The ANN comprised two hidden layers with Rectified Linear Unit (ReLU) activations, selected for their effectiveness in modeling complex non-linear relationships while mitigating vanishing gradient problems (69, 70). The first hidden layer contained 64 neurons and the second 32 neurons, with L2 regularization applied to the latter to reduce overfitting and enhance generalization (71). The output layer was configured according to prediction type: a sigmoid activation with binary cross-entropy loss for binary targets, a linear activation with mean squared error loss for continuous targets, and a softmax activation with sparse categorical cross-entropy loss for categorical targets (72, 73). Model training employed the Adam optimizer, chosen for its adaptive learning rates and favorable convergence properties (74).

### 8.2 Data stratification and preprocessing

After survival modeling and variable selection, the dataset was stratified into individuals who developed T2DM during follow-up and those who remained non-diabetic for system-level comparison. Outcome labels and time-dependent variables were excluded prior to ANN training to prevent target leakage and ensure that learned representations reflected predictor–predictor relationships rather than implicit outcome information, consistent with recommended machine learning practice (41, 75). Continuous predictors such as BMI and age were scaled to the [0,1] range using min–max normalization to improve numerical stability and convergence during training (76).

For the Cox model, variables including BMI, age, and smoking status were categorized to support hazard ratio interpretation and maintain epidemiological conventions. In contrast, for the ANN, BMI and age were kept as continuous predictors to retain informational resolution and allow non-linear relationships to be learned, while smoking status was collapsed into a binary indicator. This binarization reflects the dominant contrast in smoking-related risk, which is current or former exposure vs. no exposure. It also reduces the impact of sparse subgroups while preserving the underlying risk pattern that is most informative for ANN learning. Binary encoding and one-hot representations were selected according to the measurement scale and distribution of each variable, following standard guidance for handling categorical and ordinal predictors in neural network models (41, 77).

Dietary variables were processed according to their original UK Biobank structures and the number of meaningful levels retained following Cox model selection. Processed meat intake and beef intake were recorded on six-level ordered frequency scales. Because only the highest consumption levels (processed meat 5–6 times per week; beef ≥ 5 servings per week) showed distinct effects and the remaining categories were sparsely populated, these variables were collapsed into binary indicators for the ANN. The highest-intake category was contrasted against all lower levels, with “never” serving as the reference for ANN inputs. Collapsing sparse ordinal categories into binary contrasts is a common strategy to reduce noise and improve model stability when extreme exposure levels drive effects (41).

Bread type and cereal type differed fundamentally from the meat variables, as they represent nominal, mutually exclusive food categories rather than ordered frequency levels. Their categories reflect qualitatively distinct products rather than increasing amounts of the same item, and their distributions were well balanced across levels. Because they do not form an ordinal scale, these variables could not be meaningfully collapsed into binary exposure groups. Additionally, because bread and cereal types compete as distinct product choices rather than dose-dependent exposures, preserving their multi-category structure allowed the ANN to capture substitution patterns that would be lost under binary encoding. Preserving nominal predictors using multi-category or one-hot encoding is recommended when categories represent qualitatively distinct choices rather than ordered exposure levels, as collapsing can obscure substitution and interaction effects (41, 77).

Together, these preprocessing decisions aligned variable encoding with the underlying measurement structure of each predictor while preserving the information required for the ANN to learn meaningful non-linear patterns. This approach ensured methodological consistency between models and supported stable and interpretable comparisons across predictor types.

### 8.3 Weight extraction and feature influence quantification

Effective input to output weights were computed by summing products of connection weights along all hidden paths, providing a systemic importance measure rooted in Garson’s algorithm (78) and extended in ecological modeling (79, 80). For a two hidden layer network, the effective weight from input i to output o is defined as Equation 3:

$$W_{i,o}^{\mathrm{eff}}=\sum_{h_{1}}\sum_{h_{2}}w_{i,h_{1}}\;w_{h_{1},h_{2}}\;w_{h_{2},o},$$ (3)

where $w_{x,y}$ is the connection weight from node x to node y. The sums run over all neurons $h_{1}$ in the first hidden layer and all neurons $h_{2}$ in the second hidden layer. This provides a weight-based proxy for systemic importance by decomposing and aggregating connection weights across all paths. To improve stability and reduce overfitting, training was repeated across 10 runs with 5-fold cross-validation, yielding 50 networks per target (79). Feature influence scores were then obtained by multiplying weight matrices across layers and summarizing the values using the median over these 50 models (78, 80), a robust estimator less sensitive to outliers (81).

ANNs naturally capture joint influences among inputs through their layered nonlinear structure, meaning that the effect of any variable can depend on the values of others (82, 83). These interactions are not explicit multiplicative terms in the statistical sense but arise implicitly from the way weighted inputs propagate through the hidden layers (84). To prevent these internal dynamics from compromising interpretability, we did not inspect individual activations or paths directly. Instead, we derived effective connection weights using the Garson-based approach (78) and summarized them across cross-validated runs. This procedure aggregates all hidden-layer pathways into stable influence estimates that represent each variable’s integrated contribution after accounting for its interactions with others. As a result, the ANN can learn complex dependencies without introducing uncontrolled multiplicative artifacts, and the final influence matrices reflect consistent, interpretable, and robust joint effects.

### 8.4 Stability and coherence evaluation

To assess the robustness of these interactions, *stability* was evaluated, defined as the consistency of the interaction’s sign across multiple ANN training folds. For systematic interpretation, a four-tier classification was introduced:

- **Highly Stable:** ≥70% consistency in sign across folds
- **Moderately Stable:** 60%–69% consistency
- **Low Stability:** <60% consistency
- **Unstable:** approximately equal distribution of positive and negative signs (∼50%)

Stability provides a way to distinguish interaction patterns that are reproducible and potentially meaningful from those driven by noise or sampling variability, an approach commonly used to assess robustness of inferred relationships in neural network and ecological modeling frameworks (77, 85). In the context of behavioral coherence, lower stability could indicate more fluid or fragmented relationships between variables, whereas higher stability suggests more persistent associations. These definitions make it possible to interpret whether observed coherence patterns indicate stable behavioral structures or emerging disruption. The following section examines how coefficient values and their stability appear in the links between dietary choices and psychological states.

## 9 Results and discussion of ANN-derived influence modeling

**Figure 5:**
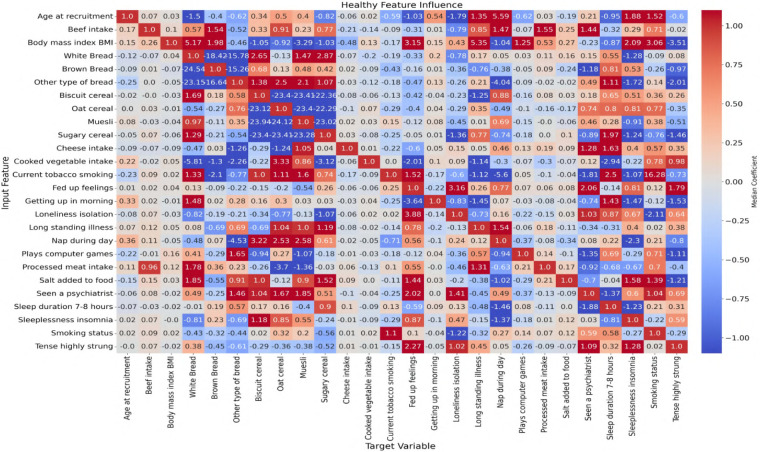
Neural network-derived feature influence matrix for healthy individuals, where darker colors show stronger positive or negative influences between variables.

**Figure 6:**
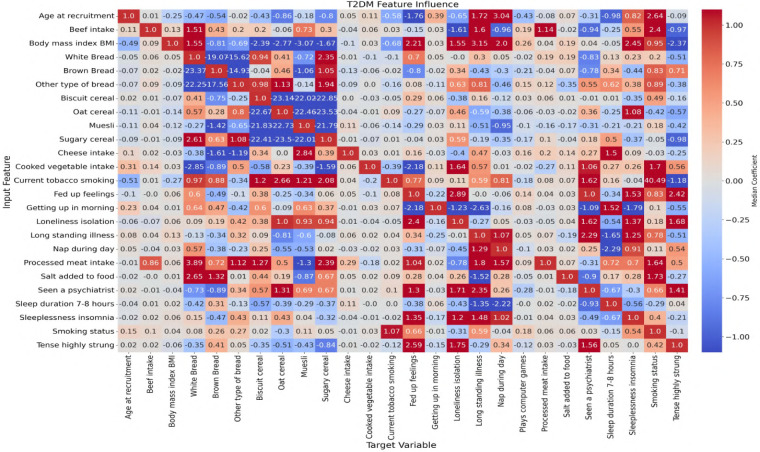
Neural network-derived feature influence matrix for T2DM individuals, where darker colors show stronger positive or negative influences between variables.

**Figure 7:**
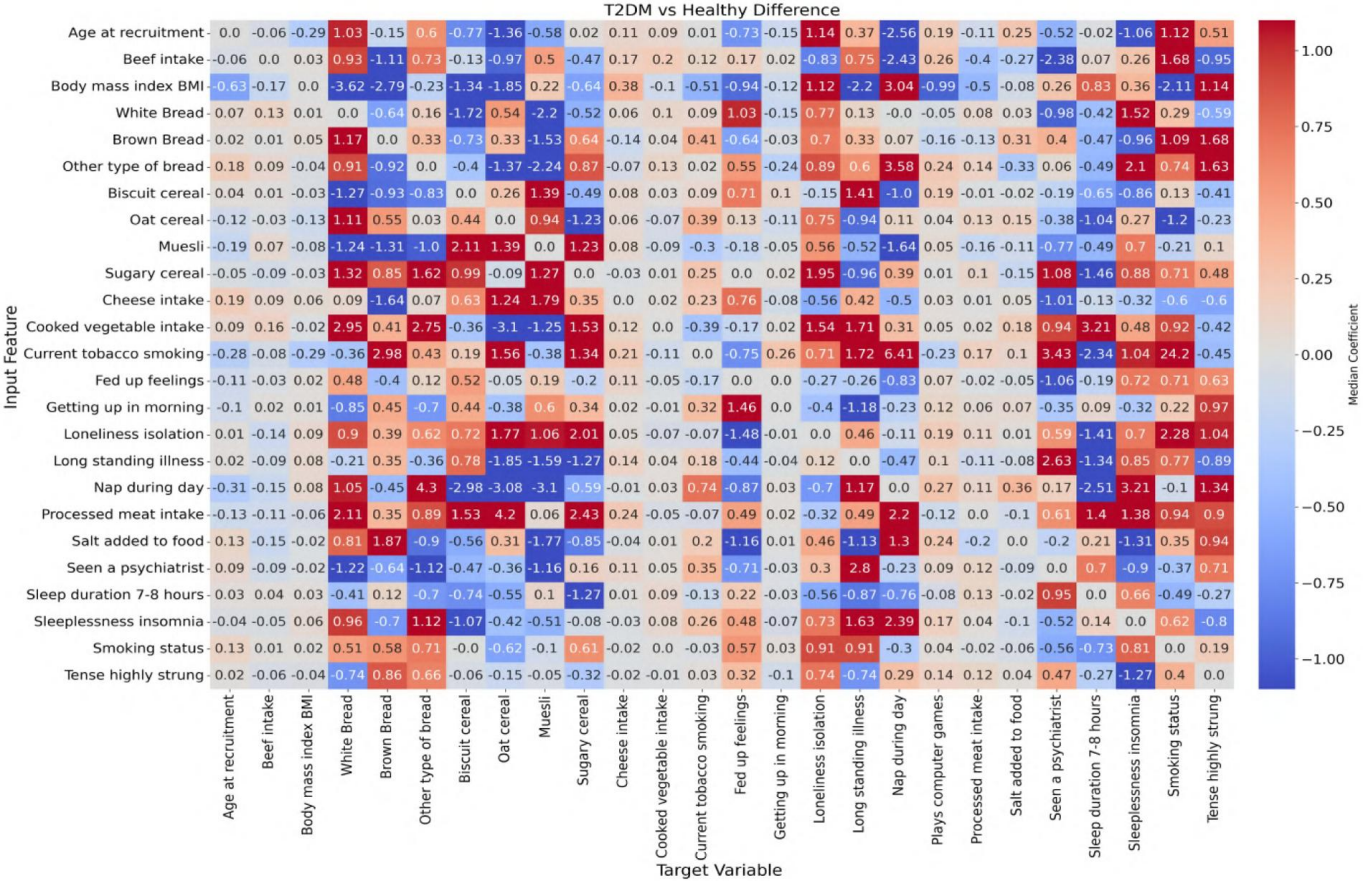
Difference matrix showing the change in feature influence between T2DM and healthy individuals (T2DM matrix minus Healthy matrix). Highlighted regions indicate variables with substantial shifts in influence relationships, potentially reflecting disrupted behavioral regulation in the diabetic state.

In this study, ANN-derived influence modeling was applied to compare Healthy and T2DM cohorts, using directional influence scores to quantify variable-to-variable associations and generate influence matrices for each group (Figures 5, 6). Subtracting the Healthy matrix from the T2DM matrix produced a Difference Matrix (Figure 7), highlighting domains in which the strength or direction of associations differed between groups. Several variables exhibited sign reversals, where associations observed in Healthy individuals changed direction in T2DM, indicating systematic reorganization of behavioral, emotional, and dietary relationships associated with disease status. These patterns are interpreted descriptively as changes in network structure rather than as evidence of causal pathways or temporal ordering (86).

To examine these systemic differences in greater detail, the following sections analyze directional associations between dietary variables and psychological states, followed by the reverse direction from psychological predictors to dietary intake. This bidirectional examination allows comparison of how food–emotion relationships are structured in Healthy individuals vs. those with T2DM.

### 9.1 Dietary-to-psychological influences

This section explores the relationship between specific food choices and psychological states in the context of T2DM. By examining how the consumption of common dietary items predicts emotional behaviors in healthy compared with T2DM individuals, the analysis highlights how chronic illness may alter the emotional impact of food.

#### 9.1.1 From comfort to coping: Emotional and demographic shifts in T2DM

In the Healthy group, certain foods serve as psychological anchors linked to comfort, regulation, or routine. However, in the context of T2DM, these same foods often become associated with emotional strain, disengagement, or maladaptive coping behaviors. Simultaneously, demographic anchors such as age and BMI, which typically guide food preferences in predictable ways, weaken or invert. This dual pattern emotional revaluation and demographic destabilization signals a broader breakdown in the structure and meaning of dietary behavior under chronic illness. The following case examples illustrate how common foods, once emotionally stabilizing and socially structured, shift in meaning and significance in the presence of metabolic strain.

Table 3 identifies variables with sign reversals, while Table 4 summarizes the effects of age and BMI on dietary intake. The β values represent directional influence scores from the neural network models, indicating the sign and relative magnitude of associations. Stability reflects the consistency of the sign across model runs shown in “%.”

**Table 3:** Neural network-derived influence scores and directional stability estimates for specific dietary items associated with psychological variables in healthy individuals and those with T2DM.

Input feature	Target variable	Healthy coeff	Healthy positive	Healthy negative	T2DM coeff	T2DM positive	T2DM negative	Coeff. difference (T2DM–Healthy)	Absolute difference
		(β)	(%)	(%)	(β)	(%)	(%)		
White bread	Fed-up feelings	−0.33	32	68	0.69	90	10	1.02	1.02
White bread	Seen a psychiatrist	0.15	60	40	−0.83	10	90	−0.98	0.98
White bread	Sleeplessness insomnia	−1.28	0	100	0.23	64	36	1.51	1.51
White bread	Tense highly strung	0.08	58	42	−0.51	28	72	−0.59	0.59
Beef intake	Seen a psychiatrist	1.43	96	4	−0.94	8	92	−2.37	2.37
Biscuit cereal	Fed-up feelings	−0.41	26	74	0.29	76	24	0.71	0.71
Biscuit cereal	Seen a psychiatrist	0.17	56	44	−0.01	50	50	−0.19	0.19
Biscuit cereal	Sleeplessness insomnia	0.50	74	26	−0.34	32	68	−0.85	0.85
Biscuit cereal	Tense highly strung	0.25	62	38	−0.15	38	62	−0.41	0.41
Brown bread	Loneliness isolation	−0.35	20	80	0.34	66	34	0.69	0.69
Brown bread	Sleeplessness insomnia	0.52	70	30	−0.43	20	80	−0.96	0.96
Brown bread	Tense highly strung	−0.97	32	68	0.71	82	18	1.68	1.68
Cheese intake	Fed-up feelings	−0.60	28	72	0.15	54	46	0.76	0.76
Cheese intake	Loneliness isolation	0.15	64	36	−0.40	22	78	−0.55	0.55
Cheese intake	Tense highly strung	0.35	76	24	−0.25	28	72	−0.60	0.60
Cooked vegetable intake	Loneliness isolation	0.09	56	44	1.63	98	2	1.54	1.54
Cooked vegetable intake	Sleeplessness insomnia	−0.21	40	60	0.26	64	36	0.48	0.48
Muesli	Loneliness isolation	−0.44	26	74	0.11	58	42	0.55	0.55
Muesli	Seen a psychiatrist	0.46	78	22	−0.30	42	58	−0.76	0.76
Oat cereal	Loneliness isolation	−0.29	28	72	0.46	84	16	0.75	0.75
Other type of bread	fed-up feelings	−0.47	34	66	0.08	52	48	0.55	0.55
Other type of bread	Loneliness isolation	−0.26	34	66	0.62	82	18	0.89	0.89
Other type of bread	Sleeplessness insomnia	−1.72	0	100	0.38	70	30	2.10	2.10
Processed meat intake	Sleeplessness insomnia	−0.67	8	92	0.70	84	16	1.37	1.37
Processed meat intake	Tense highly strung	−0.40	26	74	0.49	82	18	0.90	0.90
Salt added to food	Loneliness isolation	−0.19	40	60	0.26	68	32	0.46	0.46
Sugary cereal	Loneliness isolation	−1.36	0	100	0.59	94	6	1.95	1.95
Sugary cereal	Seen a psychiatrist	−0.89	16	84	0.18	60	40	1.07	1.07

**Table 4:** Neural network-derived influence scores for age at recruitment and BMI predicting intake of specific food items in healthy individuals and those with T2DM.

Input feature	Target variable	Healthy coeff	Healthy positive	Healthy negative	T2DM coeff	T2DM positive	T2DM negative	Coeff. difference (T2DM – Healthy)	Absolute difference
		(β)	(%)	(%)	(β)	(%)	(%)		
Age at recruitment	Beef intake	0.07	96	4	0.01	90	10	−0.0629	0.0629
Age at recruitment	Biscuit cereal	0.34	36	64	−0.43	56	44	−0.7703	0.7703
Age at recruitment	Brown bread	−0.40	10	90	−0.54	24	76	−0.1492	0.1492
Age at recruitment	Cheese intake	−0.06	12	88	0.05	94	6	0.1095	0.1095
Age at recruitment	Cooked vegetable intake	0.02	96	4	0.11	100	0	0.0904	0.0904
Age at recruitment	Muesli	0.40	76	24	−0.18	14	86	−0.5760	0.5760
Age at recruitment	Oat cereal	0.50	52	48	−0.86	6	94	−1.3583	1.3583
Age at recruitment	Other type of bread	−0.62	2	98	−0.02	22	78	0.6019	0.6019
Age at recruitment	Processed meat intake	0.03	88	12	−0.08	46	54	−0.1082	0.1082
Age at recruitment	Salt added to food	−0.19	6	94	0.07	40	60	0.2521	0.2521
Age at recruitment	Sugary cereal	−0.82	34	66	−0.80	16	84	0.0215	0.0215
Age at recruitment	White bread	−1.50	12	88	−0.47	30	70	1.0318	1.0318
Body mass index	Beef intake	0.26	100	0	0.09	96	4	−0.17	0.17
Body mass index	Biscuit cereal	−1.05	14	86	−2.39	0	100	−1.34	1.34
Body mass index	Brown bread	1.98	100	0	−0.81	8	92	−2.79	2.79
Body mass index	Cheese intake	−0.48	0	100	−0.10	2	98	0.38	0.38
Body mass index	Cooked vegetable intake	0.13	100	0	0.03	84	16	−0.10	0.10
Body mass index	Muesli	−3.29	0	100	−3.07	0	100	0.22	0.22
Body mass index	Oat cereal	−0.92	12	88	−2.77	0	100	−1.85	1.85
Body mass index	Other type of bread	−0.46	28	72	−0.69	12	88	−0.23	0.23
Body mass index	Processed meat intake	0.53	100	0	0.04	70	30	−0.50	0.50
Body mass index	Salt added to food	0.27	98	2	0.19	96	4	−0.08	0.08
Body mass index	Sugary cereal	−1.03	8	92	−1.67	0	100	−0.64	0.64
Body mass index	White bread	5.17	100	0	1.55	100	0	−3.62	3.62

White bread illustrates this dual process. In healthy individuals, its intake was modestly associated with more psychiatric consultation (β=0.15; 60% stability), but also with reduced fed-up feelings (β=−0.33; 68% stability) and fewer reports of insomnia (β=−1.28; 100% stability), suggesting a subtle comfort or stabilising role. Stability refers to the percentage of model runs in which the sign of β remained consistent (for example, β=−0.33 with 68% stability indicates a negative association in 68% of runs, whereas β=0.15 with 60% stability indicates a positive association in 60% of runs). In T2DM, BMI’s strong positive association with intake weakened markedly (1.55 in T2DM vs. 5.17 in Healthy), indicating a loss of demographic predictability. Concurrently, earlier protective emotional links diminished or disappeared, indicating that the earlier emotionally stabilizing associations are no longer present. This erosion of both demographic and emotional coherence may reflect processes such as dietary fatigue, habituation to familiar foods, and reduced emotional reward, as suggested by psychological models of eating behavior and self-regulation (87, 88). In chronic illness, white bread appears to shift from a behavior embedded in stable emotional patterns to one with reduced psychological anchoring, potentially reflecting the combined influence of dietary constraints and emotional strain.

Biscuit cereal classified as an ultra-processed food (89), shows a more decisive reversal. In the Healthy group, intake was associated with fewer fed-up feelings (β=−0.41; 74% stability), indicating a stable co-occurrence between biscuit cereal consumption and lower reported emotional strain. This was reinforced by demographic anchors: older age predicted higher intake (β=0.34; 64% stability) and BMI showed a strong negative link (β=−1.05; 86% stability), consistent with differential intake across BMI categories. However, in T2DM, the emotional association reversed to positive (β=0.29; 76% stability), indicating a positive association with emotional strain in the T2DM group. Age became a weakly stable negative predictor (β=−0.43; 56% stability), pointing to a disruption of age-related preferences, while BMI’s negative link strengthened substantially (β=−2.39; 100% stability), indicating lower reported intake at higher BMI. The combination of a robust emotional reversal and demographic destabilization observed here is consistent with a weakening of previously stable social and behavioral associations.

Beef intake had one of the largest reversals in the dataset. In Healthy individuals, it was strongly associated with psychiatric consultation (β=1.43; 96% stability), indicating a stable association between beef intake and mental health service use in this cohort. In T2DM, the association became strongly negative (β=−0.94; 92% stability), reflecting a reversal in the co-occurrence pattern observed in the Healthy group. Age associations were minimal in both groups (β=0.07 to 0.01), indicating that the change is primarily emotional rather than demographic. This reversal is consistent with a reorganization of the relationship between beef intake and psychiatric consultation in the context of chronic illness.

Processed meats followed a similar trajectory. In the Healthy group, intake was linked to reduced insomnia (β=−0.67; 92% stability), indicating a stable association between processed meat consumption and lower reported sleep disturbance. In T2DM, intake was positively associated with both insomnia (β=0.70; 84% stability) and tension (β=0.49; 82% stability). These changes may coincide with physiological states such as glycaemic disruption or inflammation (90), alongside shifts in the psychological context in which these foods are consumed. Relative to the Healthy group, processed meat intake in T2DM co-occurs more consistently with higher tension and sleep disturbance. Demographically, a weak positive age association in healthy individuals (β=0.03) shifted to a slightly negative one in T2DM (β=−0.08), indicating a gradual erosion of age-related patterns. These changes indicate a reorganization of the relationships linking processed meat intake, sleep disturbance, and tension in the context of chronic illness.

Other bread types (excluding white, brown, and wholegrain) show shifts in emotional and demographic association patterns across groups. In healthy individuals, intake was linked to lower loneliness (β=−0.26; 66% stability) and fewer fed-up feelings (β=−0.47; 66% stability), with BMI negatively associated (β=−0.46; 72% stability), indicating an association with lower loneliness and fed-up feelings in the Healthy group. In T2DM, loneliness increased with intake (β=0.62; 82% stability) and fed-up feelings approached neutrality (β=0.08; 52% stability), indicating attenuation of previously negative associations with loneliness and fed-up feelings. Age’s moderate negative link in the Healthy group (β=−0.62; 98% stability) weakened to near zero in T2DM (β=−0.02; 78%), while BMI’s negative link deepened slightly (from β=−0.46 in healthy to β=−0.69 in T2DM). Brown bread showed a similar BMI reversal, from strongly positive in healthy individuals (β=1.98; 100%) to negative in T2DM (β=−0.81; 92% stability), reflecting a weakening of previously strong BMI-related associations with intake. Together, these changes are consistent with early psychosocial shifts in chronic illness, in which demographic anchors weaken and food choices show less consistent emotional associations, coinciding with greater alignment between intake and emotional strain. Over time, such reorganization may contribute to greater variability in diet–emotion associations, with potential implications for metabolic and psychosocial regulation (88).

Sugary cereals present a partial revaluation rather than a complete reversal. In healthy individuals, intake was associated with lower loneliness (β=−1.36; 100% stability) and fewer psychiatric consultations (β=−0.89; 84% stability), indicating a consistent co-occurrence with lower reported emotional distress and mental health service use in the Healthy group. In T2DM, the associations reversed, with loneliness rising (β=0.59; 94% stability) and psychiatric consultation increasing slightly (β=0.18; 60% stability). For sugary cereals, age and BMI links were weak, with BMI remaining negatively associated in both groups. However, other cereals in the same category, such as muesli and oats, showed sharper demographic reversals. For example, muesli shifted from a positive age link in healthy individuals (β=0.40) to negative in T2DM (β=−0.18; difference −0.58), and oats from positive (β=0.50) to strongly negative (β=−0.86; difference −1.36). This suggests that the broader cereal category exhibits changes in demographic association patterns, even where sugary cereal-specific shifts are modest. These changes in sugary cereal associations do not indicate any benefit; rather, they reflect a revaluation in which such foods become more strongly associated with emotional strain in T2DM, coinciding with higher loneliness and psychiatric consultation. This shift reflects a change in the emotional context in which sugary cereals are consumed, with intake becoming more strongly associated with indicators of emotional strain in T2DM, alongside weakening demographic anchors that previously structured consumption patterns.

In conclusion, this study identifies systematic shifts in the associations between food intake, emotional indicators, and demographic variables in T2DM relative to health. Foods that, in healthy individuals, showed stable co-occurrence with lower emotional distress or predictable demographic patterning exhibit altered or reversed associations in T2DM. These changes occur alongside weakening or inversion of age- and BMI-related relationships, indicating reduced demographic structuring of dietary behavior under chronic illness. The high directional stability of these patterns across model iterations suggests that they represent consistent network-level reorganization rather than incidental variation.

Rather than remaining fixed, the emotional and demographic correlates of commonly consumed foods differ markedly between health and T2DM. Items previously associated with lower loneliness or fewer psychiatric consultations in the Healthy group show stronger alignment with indicators of emotional strain in T2DM, while demographic predictors that once anchored intake patterns become less consistent. Together, these findings point to a re-alignment of diet–emotion relationships under metabolic strain, characterized by increased coupling with emotional variables and diminished demographic patterning.

From a clinical perspective, these results underscore the importance of considering the psychological context in which dietary behaviors occur in chronic illness. Dietary guidance in T2DM may benefit from approaches that acknowledge not only nutritional composition but also the emotional and contextual factors associated with food choices. Supporting dietary strategies that maintain structure, flexibility, and psychological sustainability may help preserve adherence and well-being alongside metabolic control.

#### 9.1.2 Changes in emotional associations of healthy foods in chronic illness

While the previous discussion demonstrated that indulgent or comforting foods in healthy individuals often transitioned into stress-linked choices under T2DM, another pattern suggests that these changes extend to foods conventionally regarded as healthy, such as cooked vegetables, oats, and muesli. In Healthy individuals, these items showed neutral or protective associations with measured emotional indicators. Yet once chronic illness develops, they often show altered associations with indicators of isolation, disengagement, or emotional fatigue. This shift appears less related to nutritional composition than to the contextual factors accompanying dietary management under chronic illness.

For example, in Healthy cohort, high-fiber foods provided modest but consistent buffering. Oats were linked to reduced loneliness (β=−0.29, 72%), while muesli was associated with greater psychiatric consultation (β=0.46, 78%) and lower loneliness (β=−0.44, 74%), indicating co-occurrence with mental health service use and lower reported loneliness in the Healthy group. Cheese intake was associated with fewer fed-up feelings (β=−0.60, 72%). Together these findings suggest that “healthy” choices offered not just nutritional benefits but also were associated with lower emotional distress in the Healthy group.

In T2DM, however, the same foods exhibited altered or adverse emotional associations. Cooked vegetables, neutral in Healthy individuals (β=0.09, 56%), became strongly linked to loneliness (β=1.63, 98%), coinciding with higher loneliness in T2DM, consistent with a shift in co-occurrence patterns between dietary practices and emotional indicators. Oats reversed to predict higher loneliness (β=0.46, 84%), while muesli lost its stabilizing role: its positive link with psychiatric consultation (β=0.46, 78%) became negative (β=−0.30, 58%), and its buffering against loneliness (β=−0.44, 74%) turned mildly adverse (β=0.11, 58%). These changes indicate not only reversal but also reduced consistency in emotional association patterns, where foods previously showing neutral or protective associations exhibit less consistent emotional associations. Cheese intake also shifted from protective to mildly adverse (β=0.15, 54%), hinting at conflicted roles in T2DM, indicating heterogeneity in emotional associations, with intake linked to both neutral and adverse emotional indicators in T2DM.

In summary, ANN-derived patterns indicate that chronic illness is associated with systematic changes in the emotional and demographic correlates of foods conventionally regarded as healthy. Several items that showed neutral or protective emotional associations in the Healthy group exhibit altered or reversed associations in T2DM, with asymmetric stability across cohorts, indicating that while the direction of change is robust, its strength varies by group. These shifts occur alongside weakening or inversion of age- and BMI-related associations, suggesting reduced demographic structuring of dietary behavior under chronic illness.

Together, these findings point to a re-alignment of diet–emotion relationships under metabolic strain, characterized by stronger coupling with emotional indicators and less consistent demographic patterning. Considered alongside indulgent foods, the results indicate that both ends of the dietary spectrum show altered emotional associations in T2DM, highlighting a broader reorganization of the diet–emotion network rather than isolated effects limited to specific food categories.

From a clinical perspective, these patterns underscore the importance of considering the emotional context in which dietary changes occur in chronic illness (91). Dietary guidance in T2DM may therefore benefit from approaches that address not only nutritional composition but also the emotional and contextual factors associated with food intake, with the aim of supporting sustainable eating patterns and long-term adherence alongside metabolic control (92).

### 9.2 Psychological-to-dietary influences

Inverting the direction of influence from diet-to-emotion, this section examines how psychological states are associated with specific dietary behaviors in both healthy individuals and those with T2DM. Modeling psychological features as predictors of food intake reveals a clear pattern: emotional states, which in the Healthy group showed weaker associations with dietary behavior, exhibit stronger and less consistent associations with food intake in T2DM. The ANN-derived coefficients in Table 5 capture these shifts, showing both the magnitude and stability of associations, with particular emphasis on variables that reverse in sign between groups. A parallel pattern of change is observed when demographic variables such as age and BMI are modeled as predictors of psychological states (Table 6).

**Table 5:** Neural network-derived influence scores and directional stability estimates for psychological variables associated with specific dietary items in healthy individuals and those with T2DM.

Input feature	Target variable	Healthy coeff	Healthy positive	Healthy negative	T2DM coeff	T2DM positive	T2DM negative	Coeff. difference (T2DM - Healthy)	Absolute difference
		(β)	(%)	(%)	(β)	(%)	(%)		
Fed up feelings	Beef intake	0.02	72	28	0.00	46	54	−0.02	0.02
Fed up feelings	Biscuit cereal	−0.14	42	58	0.37	72	28	0.52	0.52
Fed up feelings	Cheese intake	−0.05	26	74	0.05	74	26	0.10	0.10
Loneliness isolation	Beef intake	0.07	78	22	−0.07	12	88	−0.14	0.14
Loneliness isolation	Biscuit cereal	−0.33	24	76	0.38	84	16	0.72	0.72
Loneliness isolation	Brown bread	−0.19	32	68	0.19	68	32	0.38	0.38
Loneliness isolation	Cooked vegetable intake	0.02	68	32	−0.04	18	82	−0.06	0.06
Loneliness isolation	Muesli	−0.13	40	60	0.93	98	2	1.06	1.06
Loneliness isolation	Oat cereal	−0.76	10	90	1.00	92	8	1.77	1.77
Loneliness isolation	Other type of bread	−0.20	38	62	0.41	86	14	0.62	0.62
Loneliness isolation	Sugary cereal	−1.07	2	98	0.93	100	0	2.00	2.00
Loneliness isolation	White bread	−0.81	10	90	0.08	54	46	0.90	0.90
Seen a psychiatrist	Beef intake	0.08	86	14	0.00	48	52	−0.08	0.08
Seen a psychiatrist	Cheese intake	−0.10	10	90	0.00	54	46	0.10	0.10
Seen a psychiatrist	Cooked vegetable intake	−0.03	20	80	0.01	62	38	0.05	0.05
Seen a psychiatrist	White bread	0.49	74	26	−0.72	16	84	−1.21	1.21
Sleeplessness insomnia	Brown bread	0.22	70	30	−0.47	14	86	−0.69	0.69
Sleeplessness insomnia	Other type of bread	−0.68	16	84	0.42	88	12	1.11	1.11
Sleeplessness insomnia	White bread	−0.81	24	76	0.15	64	36	0.96	0.96
Tense highly strung	Brown bread	−0.44	24	76	0.41	76	24	0.85	0.85
Tense highly strung	Cheese intake	0.01	60	40	0.00	44	56	−0.02	0.02
Tense highly strung	Other type of bread	−0.61	26	74	0.05	56	44	0.66	0.66
Tense highly strung	Processed meat intake	−0.08	20	80	0.02	62	38	0.11	0.11
Tense highly strung	White bread	0.38	70	30	−0.35	28	72	−0.73	0.73

**Table 6:** Neural network-derived influence scores for BMI and age at recruitment predicting psychological state variables in healthy individuals and those with T2DM.

Input feature	Target variable	Healthy coeff	Healthy positive	Healthy negative	T2DM coeff	T2DM positive	T2DM negative	Coeff. difference (T2DM – Healthy)	Absolute difference
		(β)	(%)	(%)	(β)	(%)	(%)		
Age at recruitment	Fed up feelings	−1.02	6	94	−1.75	0	100	−0.73	0.73
Age at recruitment	Loneliness isolation	−1.79	0	100	−0.65	8	92	1.14	1.14
Age at recruitment	Seen a psychiatrist	0.21	76	24	−0.30	20	80	−0.51	0.51
Age at recruitment	Sleeplessness insomnia	1.88	100	0	0.82	88	12	−1.05	1.05
Age at recruitment	Tense highly strung	−0.59	24	76	−0.08	48	52	0.51	0.51
Body mass index	Fed up feelings	3.14	100	0	2.20	100	0	−0.94	0.94
Body mass index	Loneliness isolation	0.43	68	32	1.55	100	0	1.11	1.11
Body mass index	Seen a psychiatrist	−0.22	44	56	0.03	54	46	0.26	0.26
Body mass index	Sleeplessness insomnia	2.08	100	0	2.44	100	0	0.36	0.36
Body mass index	Tense highly strung	−3.51	0	100	−2.36	0	100	1.14	1.14

In the Healthy group, these factors tend to function as stabilizing anchors, supporting predictable links between emotional states and dietary patterns. In T2DM, these relationships often weaken or reverse, indicating a loss of the demographic grounding that ordinarily contributes to behavioral stability. These changes mirror the altered patterns observed in emotional–dietary associations, suggesting that chronic illness is associated with broader reorganization of emotion–diet and demographic–psychological association patterns.

#### 9.2.1 Emotional regulation and dietary shifts in T2DM

In the Healthy group, negative emotional states showed consistent associations with lower intake of several foods, corresponding to patterns aligned with health maintenance. For example, fed-up feelings were linked to reduced intake of biscuit cereal (β=−0.14, 58% stability) and cheese (β=−0.05, 74% stability), while beef intake was essentially neutral (β=0.02, 72% stability). Loneliness was associated with reduced consumption of biscuit cereal (β=−0.33, 76% stability), brown bread (β=−0.19, 68% stability), and especially sugary cereals (β=−1.07, 98% stability). The high directional stability of these negative coefficients indicates that, in health, emotional distress consistently co-occurs with lower intake of both indulgent and staple foods. These patterns may reflect differences in appetite, restraint, or food engagement during periods of distress, without implying causal direction.

In T2DM, by contrast, these associations weaken or reverse. Fed-up feelings are now linked to higher biscuit cereal intake (β=0.37, 72% stability) and cheese intake (β=0.05, 74% stability), while loneliness is associated with greater consumption of biscuit cereal (β=0.38, 84% stability), brown bread (β=0.19, 68% stability), and sugary cereals (β=0.93, 100% stability). The largest absolute change, a Δβ=2 shift for loneliness and sugary cereals, represents a complete reversal in the direction of association, from negative to positive.

A similar contrast is observed when demographic variables are examined as predictors of psychological states (Table 6). In the Healthy group, older age was associated with lower loneliness (β=−1.79, 100% stability) and “fed-up” moods (β=−1.02, 94% stability), as well as greater psychiatric engagement (β=0.21, 76% stability). Higher BMI was linked to lower tension (β=−3.51, 100% stability) and only a mild increase in loneliness (β=0.43, 68% stability). Together, these emotional and demographic associations were organized in a consistent pattern, aligning with the stable structure observed in the Healthy group.

In T2DM, these demographic relationships show marked differences relative to the Healthy group. Higher BMI, which in health had only a mild association with loneliness and a strong tension-buffering effect, is now more strongly associated with loneliness (β=1.55, 100% stability) and has a weaker link to reduced tension (β=−2.36, 100% stability). Older age, once associated with lower loneliness and “fed-up” moods, and with higher psychiatric engagement, now shows a diminished protective association with loneliness (β=−0.65, 92% stability), is less strongly linked to lower “fed-up” moods (β=−1.75, 100% stability), and is associated with reduced psychiatric engagement (β=−0.30, 80% stability).

These concurrent changes in emotional–dietary and demographic–psychological associations indicate a shift from more consistent patterns in the Healthy group to more heterogeneous association patterns in T2DM. Instead of stable and predictable gradients, the T2DM heatmaps display irregular and sometimes inverted alignments, where previously observed age and BMI-related association patterns are weakened or reversed. As these demographic associations weaken, emotional states show less consistent alignment with dietary patterns, coinciding with greater variability in intake of energy-dense foods. In this altered landscape, demographic factors that were previously associated with more constrained mood–diet relationships are instead linked to stronger co-occurrence between negative affect and intake of high-glycemic or highly palatable foods, coinciding with patterns of emotional eating that appear more variable and less constrained (93, 94).

#### 9.2.2 Loneliness and carbohydrate intake patterns in T2DM

Loneliness emerged as one of the most pronounced points of divergence in association patterns between health and chronic illness. In the Healthy group, it was linked to lower intake of oat cereal (β=−0.76, 90% stability), muesli (β=−0.13, 60% stability), and white bread (β=−0.81, 90% stability), indicating that higher loneliness co-occurred with lower intake of carbohydrate-rich staples. In T2DM, these associations not only weakened but inverted: oat cereal (β=1.00, 92% stability), muesli (β=0.93, 98% stability), and white bread (β=0.08, 54% stability) all increased with loneliness. The most pronounced shift was observed for sugary cereals, moving from a strong negative association in healthy individuals (β=−1.07, 98% stability) to a strong positive association in T2DM (β=0.93, 100% stability), a Δβ=2.00 change.

This reversal pattern indicates that, in health, loneliness is consistently associated with lower engagement with carbohydrate-rich foods, coinciding with lower reported intake across multiple carbohydrate-rich foods. In T2DM, loneliness shows a consistent positive association with intake of high-glycemic carbohydrates, aligning with patterns commonly described as emotionally driven or compensatory eating in the literature (93, 94). Such patterns may plausibly coincide with greater glycemic variability and highlight the clustering of emotional isolation with dietary behaviors that are less aligned with metabolic stability (95).

#### 9.2.3 Sleep disruption and starch intake patterns in T2DM

In healthy individuals, insomnia is associated with reduced intake of other bread (β=−0.68, 84% stability) and white bread (β=−0.81, 76% stability), co-occurring with lower intake of staple carbohydrate foods. In T2DM, patterns diverge by food: white bread shifts to a positive association (β=0.15, 64% stability), other bread also becomes positive (β=0.42, 88% stability), whereas brown bread flips from positive in Healthy (β=0.22, 70% stability) to negative in T2DM (β=−0.47, 86% stability). This pattern indicates that, in T2DM, insomnia is associated with a shift in the direction of associations between sleep disruption and intake of specific bread types, with higher insomnia scores co-occurring with greater intake of some easily accessible, high-glycemic foods. Such co-occurring patterns highlight co-occurring patterns between sleep disruption and dietary behaviors that may have implications for glycemic regulation (91).

#### 9.2.4 Psychiatric support and breakdown of nutritional alignment

In the Healthy group, psychiatric consultation was linked to dietary choices broadly aligned with health maintenance, including higher intake of muesli (β=0.46, 78% stability) and beef (β=0.08, 86% stability). In T2DM, this coherence weakened or reversed: muesli shifted to a negative association (β=−0.30, 58% stability) and beef became neutral (β=0.00, 48% stability). The most pronounced change was for white bread, which shifted from strongly positive in the Healthy group (β=0.49, 74% stability) to strongly negative in T2DM (β=−0.72, 84% stability), a Δβ=−1.21 reversal.

Such reversals indicate that, in T2DM, psychiatric consultation shows altered associations with dietary variables relative to the Healthy group, coinciding with less consistent alignment between mental health service use and dietary patterns. In these contexts, dietary variables show weaker or inverted associations with psychiatric consultation, reflecting reduced coherence between psychological support indicators and dietary intake patterns. To highlight the complementary nature of Sections 9.1, 9.2, Table 7 summarizes these complementary dietary-to-psychological and psychological-to-dietary perspectives, highlighting how chronic illness is associated with altered bidirectional associations between dietary intake and psychological indicators.

**Table 7:** Bidirectional insights from dietary-to-psychosocial and psychosocial-to-dietary analyses, incorporating age and BMI as demographic anchors. The table highlights how chronic illness transforms the interplay between emotional meaning and regulation of food choices.

Aspect	Dietary→Psychosocial	Psychosocial→Dietary
Direction	Diet is associated with psychosocial states, considered alongside Age and BMI as demographic anchors.	Psychosocial states are associated with diet, considered alongside Age and BMI as demographic anchors.
Key question	How do foods relate to emotions, and how do Age and BMI shape these links?	How do emotions relate to eating choices, and how do Age and BMI shape these links?
Healthy pattern	Stable, intuitive links. Example: oats associate with lower loneliness; older age associates with more muesli; higher BMI shows a slight negative association with psychiatric contact.	Negative affect often suppresses intake of high GI or processed items. Example: loneliness associates with lower sugary cereal. Age and BMI provide stabilizing anchoring.
T2DM pattern	Emotional meanings often invert or fragment, and demographic anchoring weakens or reverses.	Emotional cues invert or narrow. Example: loneliness promotes high GI choices. Demographic anchoring loses stabilizing roles.
Novel insight	Chronic illness reconfigures the emotional function of foods and attenuates demographic anchoring.	Chronic illness alters the behavioral role of emotions, increasing erratic or reward-driven eating.
Coherence marker	High-stability sign flips and loss of interpretable food, emotion, and demographic patterns.	Fragmentation or inversion of emotional regulation of diet, replacing stable gradients with maladaptive patterns.
Contribution of study	Psychological revaluation of foods with reduced demographic anchoring.	Altered emotional regulation of eating, amplified by weaker demographic anchoring.
Intervention	Dietary advice should anticipate shifting emotional meanings and reduced demographic predictability.	Support should target emotional triggers and hedonic coping to prevent maladaptive eating spirals.

Taken together, these case studies show how T2DM disrupts the interconnected pathways that, in health, link psychological states, clinical support, and dietary behavior into a coherent and stabilizing framework. Social influences such as loneliness, physiological stressors like sleep disruption, and structural supports such as psychiatric contact all display a recurring pattern of weakened, reversed, or amplified dietary associations. In the Healthy group, these links typically encouraged health-supportive eating or restrained high-glycemic intake, functioning as buffers against dysregulated consumption. In T2DM, the same pathways more often direct emotional distress or clinical encounters toward variable, reward-driven eating patterns. This multi-layered disruption dismantles the regulatory scaffolding that normally anchors mood–diet relationships, leaving food choices more exposed to immediate coping needs and less tied to long-term nutritional goals, compounding the difficulty of maintaining metabolic stability.

## 10 Network cohesion and structural shifts

While the previous sections focused on directional associations between individual variables, this section examines how patterns of association across entire behavioral networks differ under chronic illness, introducing a systems-level analysis of cohesion and structural shifts using ANN-derived influence matrices to map how dietary, psychological, demographic, and lifestyle variables are interconnected. By comparing the topology of these networks in healthy and T2DM groups, the analysis identifies not only which relationships change but also how the overall structure of associations differs across health states.

### 10.1 Overview and methodological rationale

Unlike conventional model interpretation tools such as SHAP and PDPs, which assess feature importance primarily in isolation, these approaches do not capture how inter-variable association patterns shift collectively across health states (11, 12). As a result, they provide limited insight into how relationships among behavioral, psychological, and demographic variables differ between healthy and disease conditions.

To address this gap and extend the bidirectional emotional–dietary patterns described in Sections 9.1–9.2, a network cohesion framework was implemented using variable-to-variable influence matrices derived from ANN models. Directed influence networks were constructed separately for healthy and T2DM individuals, with nodes representing variables (dietary items, psychological states, demographic factors, and lifestyle behaviors) and edges representing the magnitude of positive influence between variables derived from ANN-based importance matrices. The ANN models for each group were not evaluated solely on predictive performance; instead, their internal variable-to-variable influence matrices were extracted and compared. These matrices formed the basis for the network cohesion analysis, enabling the structural reorganization of behavioral–psychological–dietary relationships to be mapped between health states. It should be noted that throughout this section, terms such as “influence,” “hub,” “driver,” and “reactivity” refer to relative network positioning and connectivity as quantified by centrality and directional metrics, rather than to causal, temporal, or behavioral mechanisms.

To characterize the systemic influence of each variable, five network centrality metrics were computed:

- In-degree: influence received from other nodes
- Out-degree: influence exerted on others
- Eigenvector centrality: connectedness to influential variables
- Betweenness centrality: extent to which a variable bridges clusters
- PageRank: composite measure balancing the quality and quantity of inbound links

**Figure 8:**
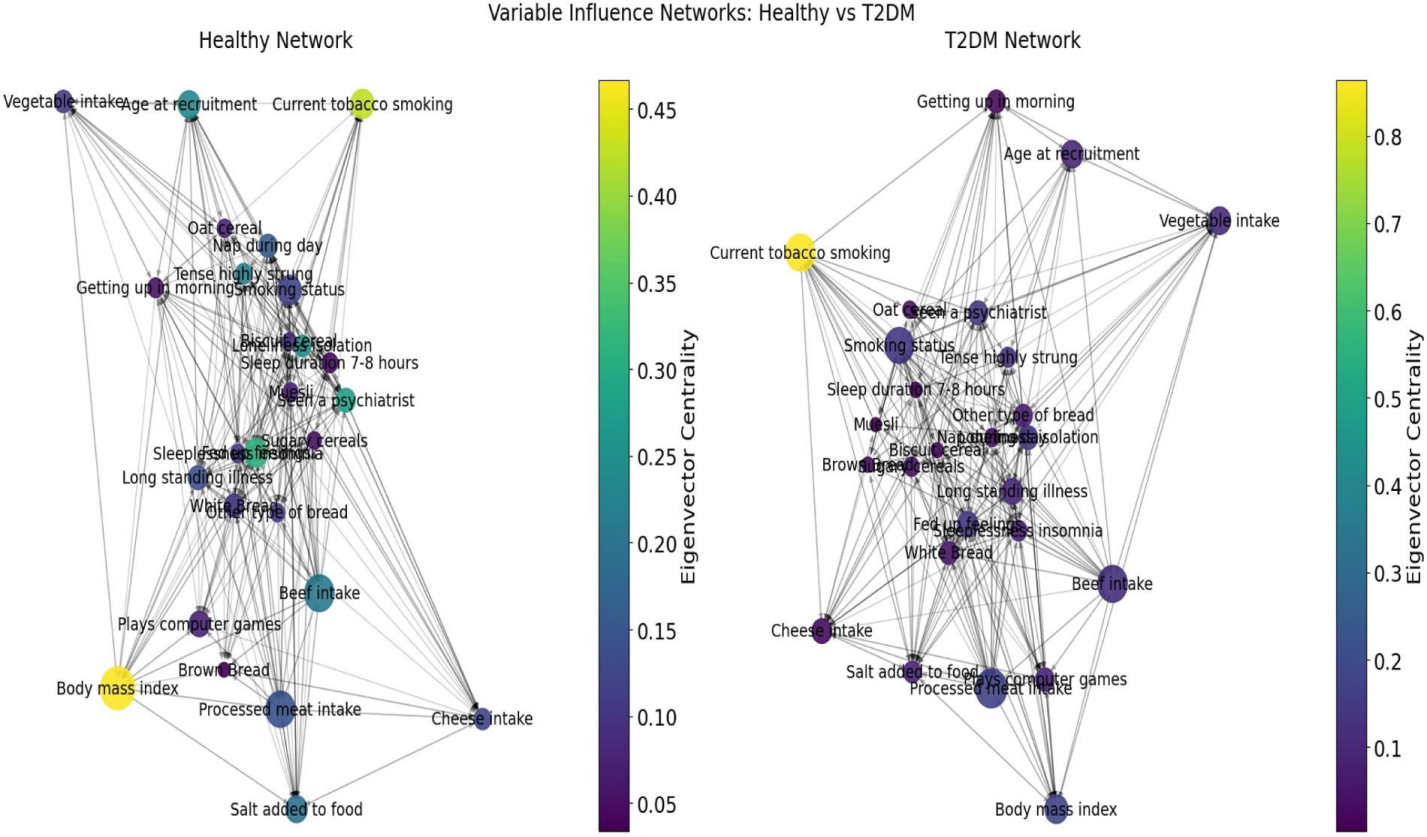
Variable influence networks for healthy and T2DM individuals. Node size represents eigenvector centrality, node color reflects relative importance, and edges indicate directional influence.

**Figure 9:**
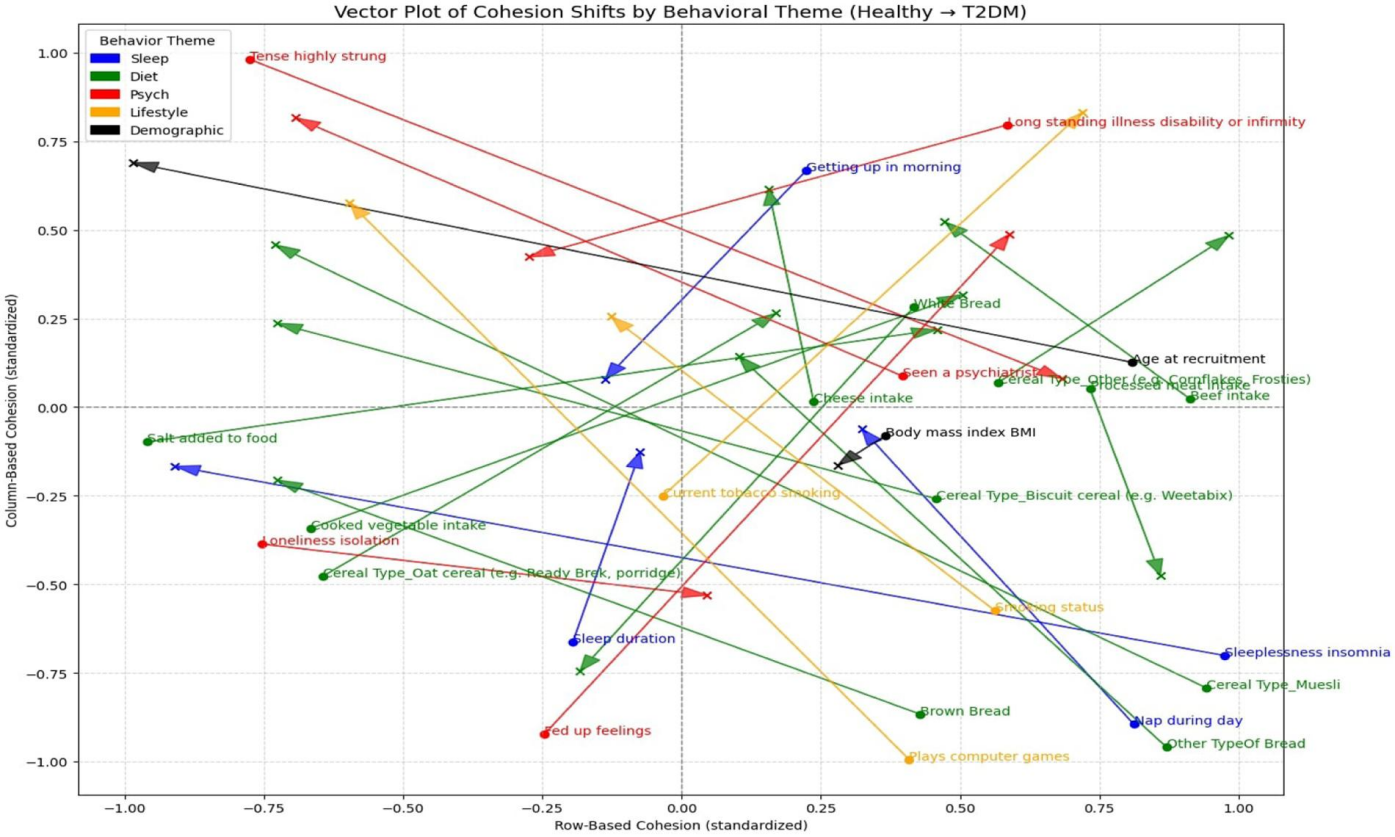
Vector shift plot of cohesion changes from Healthy to T2DM. Arrows indicate the direction and magnitude of shifts across behavioral themes.

Together, these measures characterize not only variable importance but also how variables differ in their relative positioning and connectivity within the network. Figures 8, 9 visualize network structure: node size reflects PageRank, color intensity shows eigenvector centrality, and vector shift plots illustrate cohesion changes from Healthy (base) to T2DM (arrowhead). In these plots, arrow length indicates the magnitude of change, direction distinguishes gains from losses in influence, and colors denote categories (green=dietary, red=psychological, orange=lifestyle, blue=sleep). Detailed values for PageRank, eigenvector centrality, and related metrics are provided in Table 8.

**Table 8:** Values represent centrality metrics including in-degree, out-degree, eigenvector, betweenness and PageRank for each variable, showing shifts from Healthy to T2DM networks. Results are split across two tables, with the continuation provided below.

Variable	In_degree	In_degree	In_degree	Out_degree	Out_degree	Out_degree	Eigenvector	Eigenvector	Eigenvector
	(Healthy)	(T2DM)	Δ	(Healthy)	(T2DM)	Δ	(Healthy)	(T2DM)	Δ
Age at recruitment	0.52	0.36	−0.16	0.48	0.40	−0.08	0.24	0.10	−0.14
Beef intake	0.64	0.64	0.00	0.72	0.72	0.00	0.21	0.12	−0.09
Body mass index	0.56	0.56	0.00	0.44	0.56	0.12	0.47	0.18	−0.29
Brown bread	0.40	0.32	−0.08	0.36	0.44	0.08	0.03	0.04	0.01
Biscuit cereal	0.44	0.48	0.04	0.52	0.64	0.12	0.09	0.02	−0.07
Muesli	0.48	0.20	−0.28	0.60	0.36	−0.24	0.07	0.01	−0.07
Oat cereal	0.40	0.28	−0.12	0.56	0.48	−0.08	0.08	0.05	−0.03
Sugary Cereals	0.32	0.36	0.04	0.52	0.56	0.04	0.05	0.05	0.00
Cheese intake	0.64	0.64	0.00	0.32	0.48	0.16	0.12	0.03	−0.09
Cooked vegetable intake	0.40	0.60	0.20	0.36	0.40	0.04	0.11	0.08	−0.03
Current tobacco smoking	0.44	0.72	0.28	0.28	0.52	0.24	0.43	0.87	0.44
Fed up feelings	0.64	0.56	−0.08	0.44	0.60	0.16	0.31	0.15	−0.16
Getting up in morning	0.48	0.56	0.08	0.60	0.52	−0.08	0.05	0.03	−0.03
Loneliness isolation	0.40	0.60	0.20	0.36	0.48	0.12	0.25	0.14	−0.11
Long standing illness	0.56	0.60	0.04	0.56	0.60	0.04	0.14	0.10	−0.04
Nap during day	0.52	0.52	0.00	0.52	0.48	−0.04	0.16	0.05	−0.11
Other type of bread	0.40	0.56	0.16	0.40	0.52	0.12	0.11	0.08	−0.03
Processed meat intake	0.52	0.80	0.28	0.56	0.68	0.12	0.14	0.18	0.03
Salt added to food	0.56	0.72	0.16	0.48	0.64	0.16	0.19	0.09	−0.11
Seen a psychiatrist	0.56	0.60	0.04	0.60	0.52	−0.08	0.28	0.14	−0.14
Sleep duration 7–8 h	0.52	0.32	−0.20	0.60	0.52	−0.08	0.05	0.00	−0.04
Sleeplessness insomnia	0.48	0.56	0.08	0.52	0.64	0.12	0.11	0.09	−0.02
Smoking status	0.64	0.72	0.08	0.76	0.84	0.08	0.12	0.15	0.03
Tense highly strung	0.44	0.48	0.04	0.40	0.32	−0.08	0.22	0.12	−0.10
White bread	0.52	0.56	0.04	0.56	0.60	0.04	0.10	0.05	−0.05

Variable	Betweenness	Betweenness	Betweenness	PageRank	PageRank	PageRank
	(Healthy)	(T2DM)	Δ	(Healthy)	(T2DM)	Δ
Age at recruitment	0.04	0.00	−0.04	0.05	0.04	0.00
Beef intake	0.14	0.16	0.02	0.08	0.08	0.00
Body mass index	0.00	0.09	0.09	0.11	0.05	−0.06
Brown bread	0.14	0.00	−0.14	0.01	0.01	0.00
Biscuit cereal	0.02	0.14	0.12	0.02	0.01	0.00
Muesli	0.21	0.01	−0.20	0.02	0.01	−0.01
Oat cereal	0.00	0.02	0.02	0.02	0.02	0.00
Sugary Cereals	0.05	0.03	−0.02	0.02	0.02	0.00
Cheese intake	0.16	0.13	−0.03	0.03	0.04	0.01
Cooked vegetable intake	0.16	0.16	−0.01	0.03	0.04	0.01
Current tobacco smoking	0.10	0.14	0.04	0.05	0.08	0.03
Fed up feelings	0.00	0.00	0.00	0.05	0.04	−0.01
Getting up in morning	0.52	0.30	−0.22	0.02	0.03	0.01
Loneliness isolation	0.01	0.02	0.02	0.03	0.04	0.01
Long standing illness	0.22	0.00	−0.22	0.04	0.04	0.01
Nap during day	0.05	0.16	0.11	0.03	0.02	−0.01
Other type of bread	0.00	0.08	0.08	0.02	0.03	0.01
Processed meat intake	0.04	0.08	0.04	0.08	0.09	0.01
Salt added to food	0.10	0.17	0.07	0.04	0.03	−0.02
Seen a psychiatrist	0.02	0.05	0.03	0.03	0.04	0.00
Sleep duration 7–8 h	0.00	0.07	0.07	0.02	0.01	−0.01
Sleeplessness insomnia	0.00	0.07	0.07	0.02	0.03	0.01
Smoking status	0.16	0.15	−0.01	0.05	0.08	0.03
Tense highly strung	0.02	0.03	0.01	0.03	0.03	0.00
White bread	0.04	0.00	−0.04	0.03	0.03	0.00

### 10.2 Loss of protective anchors

In the Healthy network, stabilizing variables such as age at recruitment (eigenvector=0.24, betweenness=0.04), BMI (eigenvector=0.47, PageRank=0.11), and wholegrain or high-fiber foods such as oat cereal (eigenvector=0.08) and muesli (eigenvector=0.07, betweenness=0.21) occupied central positions, often acting as bridges between dietary and emotional domains. As observed in Figure 8, these nodes appear as larger and more brightly connected in the Healthy network, reflecting their relatively higher connectivity within the Healthy network. In T2DM, these variables showed reduced centrality across multiple network metrics; BMI’s eigenvector centrality fell by −0.29 and PageRank by −0.06, but retained some bridging capacity, with betweenness rising by 0.09. Age dropped by −0.14 in eigenvector and −0.16 in in-degree, muesli declined by −0.07 in eigenvector and −0.28 in in-degree, and oat cereal by −0.03 in eigenvector. Figure 9 visually reinforces this decline, with clear leftward arrows for muesli and oat cereal, showing reduced cohesion between health states. This aligns with earlier findings indicating that variables associated with protective patterns in the Healthy group exhibit weaker emotional and structural associations in T2DM.

#### 10.2.1 Rise of maladaptive hubs

Conversely, the T2DM network showed a marked centrality gain for variables commonly linked to emotional distress and short-term behavioral patterns. As illustrated in Figure 8, current tobacco smoking shows the largest increase in centrality metrics, with the largest node size and dense outbound connections. Its eigenvector increased from 0.43 to 0.87 (Δ=0.44), in-degree from 0.44 to 0.72 (Δ=0.28), and out-degree from 0.28 to 0.52 (Δ=0.24), with PageRank also rising by 0.03. Processed meat intake gained 0.03 in eigenvector, 0.28 in in-degree, and 0.04 in betweenness, while smoking status increased by 0.03 in eigenvector and 0.03 in PageRank. Figure 9 captures these changes as arrows pointing strongly upward and outward, signifying cohesion gains. Sleep-related variables also showed increased connectivity within the network. Sleeplessness insomnia gained 0.08 in in-degree and 0.12 in out-degree, while nap during day rose in betweenness by 0.11. This structural shift indicates that, in T2DM, several lifestyle and dietary variables occupy more central network positions, with increased connectivity among tobacco use, processed food intake, and sleep-related variables.

#### 10.2.2 Directional role reversals

A notable feature of these cohesion shifts is a change in the direction of influence between variables. In healthy individuals, psychological states such as loneliness isolation (eigenvector=0.25) and fed-up feelings (eigenvector=0.31) showed higher incoming influence from dietary and lifestyle variables. In T2DM, loneliness dropped to an eigenvector of 0.14 (Δ=−0.11) but increased in in-degree (0.20) and out-degree (0.12), becoming an active driver of outgoing influence within the network, as reflected by increased out-degree and connectivity with processed food intake and tobacco use. Similarly, fed-up feelings decreased in eigenvector by −0.16 but gained 0.16 in out-degree, shifting from an emotional endpoint to a node with greater outgoing network influence. Figure 9 makes this visible: arrows for loneliness and fed-up feelings point outward and away from dietary clusters, reflecting a change in the direction of their network connections. This reversal signifies a shift from emotional regulation to reactivity, consistent with stress-induced behavioral spiral theories in chronic disease (96).

### 10.3 Systemic implications

This analysis extends the variable-specific reversals to reveal a macro-level reorganization of the network. Protective hubs lose centrality (BMI Δeigenvector=−0.29; muesli Δin-degree=−0.28), while maladaptive nodes gain strategic positions (tobacco smoking Δin-degree=0.28; processed meat Δbetweenness=0.04). Emotional states shift from primarily receiving influence to exerting greater outgoing influence (loneliness Δout-degree=0.12), altering the flow of influence within the system. Figures 8, 9 together illustrate this transformation: the Healthy network (Figure 8) is diversified, with multiple redundant protective pathways buffering fluctuations, while the T2DM network is visually clustered around fewer, maladaptive hubs. The vector map (Figure 9) shows dietary nodes (green) and psychological nodes (red) diverging from one another, with arrows pointing outward, indicating reduced coordination. In contrast, several lifestyle (orange) and sleep-related nodes (blue) move toward more integrative positions, acting as bridges between psychological and dietary domains. This marks a shift from an adaptive, multi-anchor network to a reactive, single-anchor structure that is more fragile and less able to recover from disruptions. Table 9 summarizes these dynamics, highlighting the erosion of protective anchors, the rise of maladaptive hubs, directional role reversals, and the overall reorganization from Healthy to T2DM.

**Table 9:** Summary of centrality shifts from Healthy to T2DM, showing each variable’s role, positional change, key metrics, magnitude of change (Δ), and systemic interpretation.

Variable	Role in healthy	Shift in T2DM	Key metric(s)	Δ (Healthy → T2DM)	Interpretation
Loss of protective anchors					
BMI	Central stabilizer	Hub weakened	Eigenvector, PR	−0.29, −0.06	Lost systemic link
Age	Broad stabilizer	Lower connectivity	Eigenvector, In-degree	−0.14, −0.16	Weak demographic anchor
Muesli	Bridging stabilizer	Marginalized	Eigenvector, In-degree	−0.07, −0.28	Food-mood buffer eroded
Oat cereal	Moderate stabilizer	Decline	Eigenvector	−0.03	Fiber role weakened
Rise of maladaptive hubs					
Smoking	Peripheral habit	Dominant hub	Eigenvector, In-/Out-degree	0.44, 0.28, 0.24	Drives distress, diet, lifestyle
Processed meat	Peripheral node	Bridging role	In-degree, Betweenness	0.28, 0.04	Links fat-rich diet with stress
Insomnia	Secondary connector	Stronger connector	In-degree, Out-degree	0.08, 0.12	Bridges sleep, mood, behavior
Daytime naps	Peripheral routine	Emergent bridge	Betweenness	0.11	Links disrupted routines
Directional role reversals					
Loneliness	Endpoint (in > out)	Emergent driver	Eigenvector, Out-degree	−0.11, 0.12	From outcome to driver
Fed-up feelings	Endpoint	Emergent driver	Eigenvector, Out-degree	−0.16, 0.16	Emotions drive behavior
Structural reorganization					
Network-wide	Multiple hubs	Few maladaptive hubs	Centrality dispersion	Reduced redundancy	Fragile, smoking-diet dominated

#### 10.3.1 Translational perspective

From a clinical standpoint, rising centrality in tobacco use, processed food intake, and sleep disruption identifies these behaviors as high-leverage intervention targets. Addressing them may be associated with broader changes across the emotional–behavioral network. Conversely, the loss of influence from age, BMI, and healthy food consumption suggests that traditional demographic and dietary levers may require reinforcement through psychological and behavioral support to regain their stabilizing effect. Figures 8, 9 reinforce this point by showing how structural balance is reduced and a small number of maladaptive hubs become disproportionately central within the system. Network-level monitoring, which tracks which variables act as hubs or bridges, could serve as an early warning system for deteriorating emotional resilience in chronic illness management (97, 98).

Overall, the Healthy network distributes influence across multiple adaptive nodes, ensuring redundancy and stability, whereas the T2DM network concentrates influence in fewer, maladaptive hubs. This centralization makes the system more vulnerable: changes affecting a single highly central hub, such as current tobacco smoking, may coincide with widespread alterations across multiple interconnected domains. These findings indicate that T2DM is not only a metabolic condition but a large-scale reorganization of the behavioral–emotional network toward patterns associated with greater distress and reduced adaptive health behaviors. This re-conceptualization is consistent with biomedical evidence framing T2DM as not merely metabolic but an auto-inflammatory disease driven by metabolic stress (99).

Building on these insights, the next section integrates network cohesion and centrality with the predictive power of the Cox model. This approach distinguishes early warning markers, which are predictive but peripheral, from entrenched maintenance mechanisms that are both predictive and structurally central.

## 11 Integrating survival analysis with network cohesion shifts

While Section 10 examined the structural reorganization of the behavioral–psychological–dietary network using centrality and cohesion metrics, and earlier sections evaluated variable-level predictive power via Cox proportional hazards models, these approaches offer complementary but distinct perspectives. The Cox model identifies variables with statistically significant hazard ratios (HRs) for T2DM onset, quantifying their independent contribution to disease risk or protection over time. In contrast, the network cohesion and centrality analysis captures each variable’s systemic role, whether as a hub, bridge, or peripheral node, and how that role changes from the healthy to the T2DM state. It should be noted that comparisons in this section focus solely on shared key variables and the patterns or directions of associations (e.g., hazard ratio trends, cohesion metrics) rather than direct numerical matching.

Here, the two analytical strands are integrated to identify variables that are both statistically robust predictors of T2DM and structurally central within the reorganized behavioral network. This combined view reveals not only which variables predict disease, but also how they function within the broader system once the disease has developed. Such dual-impact variables, predictive in the Cox model and central in the network, are likely to act as core disease maintenance mechanisms. Conversely, variables that are predictive but peripheral may represent early warning markers without sustained systemic influence. Identifying this distinction is critical because T2DM is not simply the sum of isolated risk factors; it is a systemic reconfiguration of behavioral, emotional, and dietary relationships, where the position and role of variables within the network can influence both disease onset and progression.

### 11.1 Concordance between hazard ratios and network roles

Several variables that emerged as strong risk factors in the Cox model also gain network centrality in T2DM, suggesting that their predictive power is reinforced by their integration into maladaptive feedback loops.

Current tobacco smoking (HR=1.34, 95% CI: 1.14–1.58, p<0.001), Δeigenvector=0.44, Δin-degree=0.28, ΔPageRank=0.03. The magnitude of its centrality increase is unmatched in the network, transforming it from a peripheral lifestyle behavior in the Healthy group into the dominant structural hub in T2DM. Tobacco’s rise likely reflects its dual role as both a coping mechanism for emotional distress and a behavior with direct metabolic effects, such as increased insulin resistance and inflammation (100). The network evidence shows it is not simply co-occurring with other high-risk habits but occupying a highly connected position linking them, linking psychological distress nodes with high-calorie dietary patterns and other lifestyle risk behaviors.

Processed meat intake (5–6 times/week) (HR=1.18, 95% CI: 1.01–1.38, p=0.03), Δeigenvector=0.03, Δbetweenness=0.04. While its hazard ratio is smaller than tobacco’s, its rise in betweenness indicates that processed meat has moved into a more strategic bridging position, connecting dietary risk clusters with psychological states. This pattern suggests that in T2DM, processed meat may serve as a link between high-salt, high-fat eating patterns and stress-related behaviors. Changes in intake may therefore be associated with altered connectivity in these links, potentially corresponding to weaker coupling between dietary risk and emotional strain.

Adding salt to food was associated with a higher risk of T2DM (HR=1.05, 95% CI: 1.02–1.08, p<0.001). This behavior was accompanied by a decrease in eigenvector centrality (−0.11) and an increase in betweenness centrality (0.07). The combination of a drop in eigenvector and a rise in betweenness suggests a role shift: salt is no longer widely connected to many influential nodes, but instead becomes a selective connector linking specific high-risk dietary patterns (such as processed meats and refined carbohydrates) with emotional distress. This more targeted but potent positioning may make salt a contributor to narrower, high-impact feedback loops in the T2DM network. In practical terms, salt transitions from being a general dietary factor to a specialized bridge that helps connect unhealthy eating patterns with mood-related vulnerabilities, meaning small changes in this link could have disproportionate effects on breaking certain diet-emotion cycles.

### 11.2 Protective factors losing influence

In contrast to variables that become central hubs in the T2DM network, several protective factors identified in the Cox model lose systemic importance once the disease emerges. This pattern suggests that while these variables may buffer against disease onset, they do not retain coordinating influence after behavioral and emotional systems reorganize around chronic illness. Their declining centrality signals the erosion of adaptive scaffolding that typically anchors healthy behavior.

While loneliness (HR=1.14, 95% CI: 1.07–1.21, p<0.001) exhibits a strong hazard ratio, it remains relatively peripheral in the T2DM network, with a decline in eigenvector centrality (Δeigenvector=−0.11) but a rise in out-degree (Δout-degree=0.12). This shift reflects greater influence over a select set of maladaptive behaviors, including processed food intake, tobacco use, and sleep disruption, rather than coordination across the whole system. Such patterns suggest that loneliness functions more as an early warning marker and targeted behavioral driver, valuable for early detection and prevention, rather than as a central modulator of chronic disease dynamics once the condition is established.

More notably, variables that once acted as structural stabilizers in healthy individuals lose integrative function in T2DM. BMI (25–29.9) (HR=0.49, 95% CI: 0.39–0.60, p<0.001), a highly central node in the Healthy network (eigenvector=0.47), drops sharply in centrality (Δeigenvector=−0.29), indicating a disconnect between weight status and coordinated diet or activity. Similarly, muesli (HR=0.57, 95% CI: 0.52–0.63, p<0.001), once a bridge linking wholegrain intake to emotional regulation, loses systemic role (Δeigenvector=−0.07; Δin-degree=−0.28), suggesting erosion of food–mood synchrony. Cheese intake (HR=0.94, 95% CI: 0.92–0.96, p<0.001) also declines in influence (Δeigenvector=−0.09), showing that even protein-rich, stabilizing foods no longer anchor adaptive behavior.

At the same time, certain emotional states that were once peripheral evolve into more influential roles, shifting from predominantly receiving influence to exerting greater outgoing network influence in T2DM. Loneliness (Δeigenvector=−0.11, Δout-degree=0.12), for example, shifts from being a downstream emotional consequence in healthy individuals to a behavioral driver, influencing smoking, sleep disruption, and increased processed food consumption. Similarly, insomnia (HR=1.06, 95% CI: 1.00–1.12, p=0.02), with increased betweenness (Δbetweenness=0.07), emerges as a cross-domain bridge linking emotional distress to routine disruption. Even with a moderate hazard ratio, its systemic position suggests that sleep-related variables are associated with multiple behavioral domains across the network. These shifts imply that emotional dysregulation not only reflects T2DM burden but increasingly shapes behavioral risk, sustaining feedback loops that reinforce disease.

These changes indicate a breakdown in the previously stable health-supporting system that helped maintain resilience. In a healthy state, certain foods and physical traits such as a moderate BMI, high-fiber cereals, or protein-rich items like cheese not only provided nutritional value but also served as behavioral anchors. They helped connect diet, mood, and daily routines in consistent and reinforcing ways. In individuals with T2DM, the weakening of these anchors leads to a more fragmented network, where fewer elements work together to support healthy behaviors across different aspects of life. Bringing back these components, not just as changes to diet but as ways to reconnect emotional and lifestyle patterns, may be associated with partial restoration of network coherence and support overall functional resilience.

### 11.3 Integrated insights for intervention

This integration highlights three categories of intervention relevance, offering a framework for prioritizing prevention and management strategies that account for both predictive strength and systemic influence:

- **Dual-priority targets:** Tobacco smoking and processed meat intake are both statistically significant predictors and structurally central in the T2DM network. Interventions targeting these behaviors may yield compounding benefits, simultaneously disrupting multiple maladaptive pathways. For example, smoking cessation programs paired with stress management could break both metabolic and psychosocial feedback loops, while reducing processed meat intake could disintegrate bridges that connect poor diet with emotional dysregulation.
- **Structural stabilizers:** Variables such as BMI, muesli, and cheese appear to lose their network anchoring roles in T2DM. While protective in the healthy state, they become disconnected from broader behavioral coordination in disease. This suggests a need to not only promote these variables as individual behaviors, but to reintegrate them into routines that support emotional and lifestyle coherence. For instance, reintroducing high-fiber cereals or protein-rich foods in structured meal plans, combined with behavioral reinforcement (e.g., consistent eating schedules, mood tracking), may help restore their stabilizing effects and enhance network resilience.
- **Emergent drivers:** Loneliness and insomnia exhibit modest hazard ratios but gain cross-domain influence in T2DM, positioning them as under-recognized behavioral levers. Their rising out-degree and betweenness suggest they no longer merely reflect poor health but actively shape it. Addressing these factors early through social support programs, sleep hygiene interventions, or integrated mental health services may interrupt self-reinforcing cycles that sustain chronic disease. These variables highlight the importance of treating emotional states not as secondary concerns, but as primary targets for system-level recovery.

In summary, the three categories above capture the distinct ways variables shape T2DM dynamics. This concise synthesis links statistical significance from the Cox model with structural influence from the network analysis, providing a clear reference for prioritizing prevention and management strategies. Overall, these findings reinforce that T2DM is not merely a collection of independent risk factors but a systemic reorganization of the behavioral–emotional network. In this reconfiguration, certain high-risk behaviors emerge as structural hubs, stabilizing factors lose influence, and some emotional states shift into active drivers of behavior. Interventions that target both the metabolic impact of these variables and their network role may be more effective in restoring resilience and breaking self-sustaining cycles of distress and maladaptation. Focusing on such dual-impact variables may offer a path toward interventions that are both clinically relevant and systemically transformative. To consolidate these findings, Table 10 summarizes the most influential variables by both predictive power and systemic network role, grouping them into intervention-relevant categories.

**Table:** TABLE 10 Key behavioral features in T2DM integrating predictive strength and network role. Variables are grouped by intervention relevance.

Category	Feature	Why it is influential?
Dual-priority targets (High risk & high centrality in T2DM)	Tobacco smoking	Highest centrality rise (Δeigenvector 0.44, Δin-degree 0.28) and strong risk (HR 1.34). Becomes dominant hub linking emotional distress with poor diet and other lifestyle risks. Disrupting it could destabilize multiple maladaptive pathways.
	Processed meat intake (5–6 times/week)	Moderate risk (HR 1.18) but gains bridging role (Δbetweenness 0.04). Connects unhealthy eating patterns with stress-related behaviors, making it a strategic dietary-psychological link.
	Salt added to food	Modest risk (HR 1.05) but shifts to selective connector (Δbetweenness 0.07). Links high-risk dietary patterns with mood vulnerabilities; small reductions could break feedback loops.
Structural stabilizers (Protective in healthy state but lose coordination in T2DM)	Muesli	Protective (HR 0.57) and formerly a key bridge (Δin-degree −0.28). Loss of influence in T2DM signals breakdown of food and mood coordination, and reintegration could restore stability.
	Cheese intake	Protective (HR 0.94) but loses centrality (Δeigenvector −0.09). Previously anchored dietary-psychological stability, suggesting strategic reintroduction could rebuild resilience.
	BMI (25–29.9)	Strongly protective (HR 0.49) in healthy state but large drop in centrality (Δeigenvector −0.29) in T2DM, indicating disconnection from coordinated health behaviors post-diagnosis.
Emergent drivers (Modest risk but gain cross-domain influence)	Loneliness isolation	Strong risk (HR 1.14) and increases influence over specific maladaptive behaviors (Δout-degree 0.12) despite lower centrality. Acts as an early warning marker and driver of smoking, poor diet, and sleep disruption.
	Sleeplessness insomnia	Moderate risk (HR 1.06) but rises in bridging capacity (Δbetweenness 0.07). Links emotional distress to routine disruption, making it a cross-domain intervention point.

## 12 Conclusion & future directions

This study provides the integrated analysis linking longitudinal T2DM risk prediction with network-level reorganization of behavioral, dietary, and psychological factors. By combining Cox proportional hazards modeling with network cohesion and centrality metrics, this study identify not only variables that predict T2DM onset but also those that become structurally central to the disease’s maintenance once established. The findings reveal a marked shift from a diversified, multi-anchor network in healthy individuals to a concentrated, hub-dominated structure in T2DM, where maladaptive behaviors such as tobacco smoking and processed meat intake assume coordinating roles. Protective factors such as high-fiber cereals, moderate BMI, and protein-rich foods lose their systemic influence, while emotional states like loneliness and insomnia shift from predominantly downstream positions to more influential roles within the behavioral network. These insights underscore that T2DM is not merely a metabolic disorder but a wholesale reprogramming of the behavioral-emotional system, sustaining cycles of distress and maladaptation.

### 12.1 Future directions

Future research should expand this integrated framework across diverse populations and clinical contexts to test the universality of these network shifts. Longitudinal network tracking, incorporating repeated psychological, dietary, and metabolic assessments, could illuminate the temporal dynamics by which protective hubs erode and maladaptive hubs consolidate. Incorporating additional domains such as physical activity, medication adherence, and social determinants of health could enrich network resolution. Furthermore, causal modeling and intervention simulations could assess whether targeted disruption of central maladaptive hubs, such as tobacco use or processed meat consumption can re-establish distributed protective architectures. Integrating wearable sensor data and digital health platforms offers another pathway for real-time detection of emerging network vulnerabilities, potentially enabling earlier and more tailored interventions. The present findings have several actionable implications:

**For policymakers:** Prevention strategies should move beyond single-factor risk reduction toward system-aware interventions that address behavioral clusters and feedback loops. National dietary guidelines and anti-smoking campaigns could be restructured to target high-centrality risk behaviors jointly, maximizing downstream benefits. Policies should also promote access to healthy, high-fiber foods while supporting community-based programs that strengthen social connectedness, addressing loneliness as a health risk.

**For clinicians:** Network-informed screening can help identify not only patients at high metabolic risk but also those whose behavioral patterns suggest systemic fragility. Incorporating simple, validated measures of emotional states (e.g., loneliness, sleep disruption) alongside traditional biomarkers could improve risk stratification. Intervention planning should pair metabolic management with behavioral restructuring, reintroducing lost protective anchors and reducing the influence of central maladaptive hubs.

**For researchers:** Network-based methodologies should be applied to intervention trials to track how behavioral-emotional architectures respond to treatment. Such designs could clarify whether restoring protective hubs translates into sustained metabolic improvement.

**For public health practitioners:** Campaigns should recognize that emotional distress and maladaptive habits often co-evolve. Integrating mental health services with dietary counseling and smoking cessation programs may yield greater impact than addressing each in isolation.

By re-framing T2DM as a system-level reorganization rather than a linear accumulation of risk factors, this study provides a conceptual and methodological template for precision prevention and integrated care. The next frontier will be to translate these network signatures into actionable tools that guide individualized and community-wide interventions, with the dual aim of reducing disease incidence and dismantling entrenched patterns of maladaptation.

## Data Availability

The data analyzed in this study is subject to the following licenses/restrictions: The UK Biobank resource is not publicly available due to participant privacy and data governance restrictions. However, it is accessible to bona fide researchers worldwide through a formal application process. Researchers must apply via the UK Biobank Access Management System (https://www.ukbiobank.ac.uk) and obtain approval for health-related research that is in the public interest. Access is granted under a Material Transfer Agreement, with restrictions including non-identification of participants, non-sharing of raw data, and use only for the approved research purposes. Requests to access these datasets should be directed to https://www.ukbiobank.ac.uk.
